# Mathematical modeling of tuberculosis using Caputo fractional derivative: a comparative analysis with real data

**DOI:** 10.1038/s41598-025-97502-5

**Published:** 2025-04-12

**Authors:** Sanjay Bhatter, Sangeeta Kumawat, Sunil Dutt Purohit, D. L. Suthar

**Affiliations:** 1 Department of Mathematics, Malaviya National Institute of Technology Jaipur, Jaipur, India; 2 Department of HEAS (Mathematics), Rajasthan Technical University, Kota, India; 3 Department of Mathematics, Wollo University, Dessie, Amhara Ethiopia; 4 Department of Mathematics, Saveetha School of Engineering (SIMATS), Thandalam, Chennai, Tamil Nadu 600124 India

**Keywords:** Tuberculosis, Caputo fractional operator, Parameter estimation, Sensitivity analysis, Numerical simulations, Computational biology and bioinformatics, Mathematics and computing

## Abstract

In this study, we investigate an epidemiological model for tuberculosis in China using the Caputo fractional-order derivative. To ensure dimensional consistency, we appropriately adjust model parameters to maintain uniform units. The mathematical properties of the model, including the non-negativity, boundedness, existence, and uniqueness of its solutions, are thoroughly examined and established. Sensitivity analysis, based on the basic reproduction number, is performed to evaluate the impact of critical parameters on disease dynamics, and additional insights are provided through 3D mesh and contour plots, which illustrate how key parameters influence tuberculosis transmission. We estimate the model parameters, including the fractional-order derivative, and determine that the optimal fractional order, which best fits the real data, is approximately 0.93. Numerical simulations are performed using the Adams–Bashforth–Moulton method. By utilizing the root mean square error (RMSE) metric, the fractional-order model demonstrates an efficiency improvement of approximately 28.5% compared to its integer-order counterpart, highlighting the superior accuracy of fractional-order models in describing tuberculosis transmission dynamics. These findings underscore the significance of fractional-order models in epidemiological analysis. They provide a more refined approach for modeling infectious diseases and aiding in public health decision-making.

## Introduction

Tuberculosis (TB) is a highly transmissible disease caused by the bacterium Mycobacterium tuberculosis. It primarily targets the lungs, but in certain cases, it can also affect other organs of the body, such as the brain, kidneys, skin, and spine.^1^ TB is not a new disease; it has been known to humankind for centuries, with historical records tracing its existence back to ancient Egypt, China, and India. The disease is primarily transmitted through airborne droplets containing TB bacteria, which are released when an infected individual coughs, sneezes, speaks, or spits. Family members, friends, and close contacts of infected individuals are at high risk of contracting the disease by inhaling these bacteria. Of those infected with TB bacteria, approximately 10% develop an active TB infection, while the remaining 90% have latent TB. Individuals with latent TB do not exhibit symptoms and cannot spread the infection. Active TB, on the other hand, is characterized by symptoms such as a persistent cough lasting more than three weeks, fever, chest pain, coughing up blood, night sweats, fatigue, and unintentional weight loss. TB diagnosis is typically conducted through skin or blood tests. Individuals with compromised immune systems, such as those with HIV or diabetes, are at a higher risk of developing active TB. The World Health Organization (WHO) reported in 2013 that there were 8.6 million new cases of TB and 1.3 million deaths in 2012, with approximately 320,000 of these cases involving individuals infected with HIV. A 2018 WHO report indicated that about one-third of the global population is infected with TB, highlighting the disease’s continued prevalence as a public health crisis. China and India bear the highest TB burden globally, contributing 12% and 26% of cases, respectively, as TB continues to rank among the top 10 infectious diseases worldwide. In spite of various measures to combat the disease, such as Bacillus Calmette-Guerin (BCG) vaccination, antimicrobial chemotherapy, and antiretroviral therapy, TB continues to pose a significant health challenge in numerous high-incidence areas, including China. The emergence of drug-resistant TB strains and the spread of HIV/AIDS have exacerbated this challenge in countries like China, where TB prevalence has increased over the last two decades.

Epidemiological studies are crucial for understanding the societal impacts of infectious diseases, and mathematical modeling has emerged as a vital tool in this regard. Mathematical models are extensively used across disciplines, including natural sciences, engineering, and social sciences, to explain complex systems, analyze nonlinear processes, and predict behavior. In disease modeling, these approaches help investigate infection dynamics, estimate key parameters, and simulate scenarios to understand disease spread and control measures. TB modeling has seen significant progress over the years.^2,3^ Waaler et al.^4^ first modeled TB transmission, while subsequent researchers extended their work. Yang et al.^5^ incorporated incomplete treatment and explored fast and slow TB transmission. Okuonghae^6^ examined the effect of genetic heterogeneity on the spread of TB. Zhang et al.^7^ introduced a model with hospitalized and non-hospitalized classes using China TB data. Al-Hdaibat et al.^8^ investigated a tuberculosis model incorporating four distinct control strategies: vaccination, awareness campaigns, screening, and pathogen clearance, demonstrating their combined impact on disease mitigation.

Traditional models based on ordinary differential equations (ODEs) are often limited in capturing the complexities of biological systems. Fractional-order differential equations (FODEs), which generalize ODEs by allowing non-integer derivatives, provide a more comprehensive framework for studying these dynamics.^9–12^ Fractional derivatives account for memory effects, capturing long-term dependencies and non-local behaviors that integer-order models cannot address^13^. The use of fractional-order derivatives in modeling infectious diseases offers several advantages . Unlike integer-order derivatives, fractional derivatives account for the history of the system, enabling a more accurate representation of diseases with long-term effects, such as TB.^14–16^ Fractional-order models can also describe a wide range of dynamics, from simple to complex behaviors, by adjusting the order of the derivative. For instance, a derivative of order 1 may represent straightforward disease transmission, while a derivative of order 0.5 can capture more intricate patterns. Fractional-order models provide a continuous transition between integer-order models, offering better fits for empirical data. They are particularly suited for diseases with multiscale dynamics, where processes such as pathogen replication and immune response operate on different time scales. Additionally, they can model anomalous diffusion processes, such as non-local movements and long-tail distributions, which are often observed in disease transmission.

In recent years, fractional calculus and its wide-ranging applications have gained significant attention in various fields, including biology, finance, geology, thermodynamics, and fluid dynamics. Its ability to incorporate memory and hereditary effects provides a more comprehensive understanding of dynamic systems compared to the localized behavior of integer-order derivatives, as extensively documented in the literature.^17–21^ Among these applications,^22^ investigates the dynamics of an interacting phytoplankton species model using a fractional-order operator, demonstrating how memory effects influence the stability of equilibrium points. In,^23^ fractional derivative operators are employed to examine chaotic behavior in a financial system. Similarly,^24^ presents a fractional-order cancer treatment model that explores drug-targeting strategies through nanotechnology, emphasizing innovative approaches for improving drug delivery efficiency. Khirasiya et al.^25^ analyze a fractional-order rat bite fever model, illustrating how these models offer greater flexibility in capturing memory effects and disease progression for specific datasets. Dasumani et al.^26^ explore a fractional-order fishery resource model in the presence of predators, incorporating the Crowley-Martin functional response to better describe ecological interactions. Further, Zhang et al.^27^ formulate a fractional-order model to study the transmission dynamics of tuberculosis in a Pakistani city, highlighting the advantages of fractional calculus in epidemiological modeling. In,^28^ researchers extend an integer-order COVID-19 model to its fractional counterpart, comparing their results against actual data. Their findings reveal that a fractional-order model with a derivative order of 0.98 yields more accurate predictions than the classical integer-order approach. Qureshi et al.^29^ investigate a dengue fever outbreak using three distinct fractional-order derivative operators. Their analysis indicates that these operators provide superior efficiency compared to traditional integer-order models. The growing recognition of fractional calculus and its success in modeling real-world phenomena serve as the motivation for this study.^30,31^ Building upon this foundation, our research aims to analyze an integer-order mathematical model for tuberculosis by incorporating the Caputo fractional-order derivative. Through this approach, we seek to demonstrate how fractional-order models achieve better alignment with real data compared to their integer-order counterparts.

The structure of the paper is as follows: Section “Preliminaries” provides key definitions and concepts in fractional calculus. Section “Model formulation” introduces the proposed Caputo fractional-order TB model. Section “Model analysis” discusses the positivity, existence, boundedness and uniqueness of the model’s solutions. Section “Parameter estimation” presents sensitivity analysis of model parameters. Section “Sensitivity analysis” outlines the implementation of the model using TB data, with parameter estimation performed via MATLAB’s lsqcurvefit function. Section “Numerical simulation and discussion” offers numerical simulations using the predictor–corrector scheme, and Section “Conclusion” concludes the study with remarks on the findings and their implications.

## Preliminaries

### Definition 2.1

The fractional integral operator in the Riemann–Liouville sense, for a function $f:(0,\infty ) \rightarrow {\mathbb {R}},$ is expressed for an order $\varphi >0$ as^32^

1 $$\begin{aligned} ^{RL} _{0}{\mathfrak {I}}^{\varphi }_{t} f(t) = \dfrac{1}{\Gamma (\varphi )} \int _{0}^{t} (t-\xi )^{\varphi -1} f(\xi ) d\xi , \end{aligned}$$

here, $\varphi >0$ and $\Gamma (.)$ is a Gamma function.

### Definition 2.2

The Riemann–Liouville fractional derivative operator for a function $f:(0,\infty ) \rightarrow {\mathbb {R}}$ of order $\varphi >0$ is given as^32^

2 $$\begin{aligned} ^{RL} _{0}{\mathfrak {D}}^{\varphi }_{t} f(t)= {\left\{ \begin{array}{ll} \dfrac{1}{\Gamma (n-\varphi )} \bigg (\dfrac{d}{dt}\bigg )^{n} \int _{0}^{t} (t-\xi )^{n-\varphi -1} \, f(\xi )\, d\xi ; \ \ \ \ 0 \le n-1<\varphi < n, \\ \\ \bigg (\dfrac{d}{dt}\bigg )^{n} f(t) ; \ \ \ \ \ \ \varphi =n, n \in {\mathbb {N}}. \end{array}\right. } \end{aligned}$$

### Definition 2.3

The Caputo fractional derivative of order $\varphi$ for a function $f:(0,\infty ) \rightarrow {\mathbb {R}}$ is expressed as follows:^32^

3 $$\begin{aligned} ^{C} _{0}{\mathfrak {D}}^{\varphi }_{t} f(t)= {\left\{ \begin{array}{ll} \dfrac{1}{\Gamma (n-\varphi )}\int _{0}^{t} \dfrac{f^{n}(\xi )}{(t-\xi )^{\varphi - n +1}} \, d\xi ; \ \ \ \ 0 \le n-1<\varphi < n, \\ \\ \bigg (\dfrac{d}{dt}\bigg )^{n} f(t); \ \ \ \varphi =n, n \in {\mathbb {N}}. \end{array}\right. } \end{aligned}$$

### Definition 2.4

The Laplace transform (LT) of the Caputo operator for *f*(*t*) of order $\varphi >0$ is represented as:^33^

4 $$\begin{aligned} {\mathcal {L}}\bigg [^{C} _{0}{\mathfrak {D}}^{\varphi }_{t} f(t)\bigg ]= s^{\varphi } F(s)-\sum _{k=0}^{n-1} f^{k}(0) s^{\varphi -k-1}. \end{aligned}$$

### Definition 2.5

The two-parameter Mittag-Leffler function, denoted as $E_{m,n} (z)$ is described as^34^

5 $$\begin{aligned} E_{m,n} (z)= \sum _{k=0}^{\infty } \dfrac{z^{k}}{\Gamma (m k + n)}; m,n>0, \end{aligned}$$

and the Laplace transform of function $t^{n-1} E_{m,n} ( \pm at^{m})$ is described as:^33^

6 $$\begin{aligned} {\mathcal {L}} \bigg [t^{n-1} E_{m,n} (\pm a t^{m})\bigg ] =\dfrac{s^{m-n}}{s^{m} \mp a}. \end{aligned}$$

## Model formulation

In this study, the integer-order TB model^35^ is extended to a fractional-order model by incorporating the Caputo fractional derivative. The model categorized the host population into four main epidemiological groups. The susceptible group *S*(*t*) includes individuals who are at risk of infection. The exposed group *A*(*t*) consists of individuals who have been exposed to the infection but are not yet actively infectious. The infected group *I*(*t*) includes those currently infected and capable of spreading the disease, while the recovered group *R*(*t*) comprises individuals who have overcome the infection. To investigate the influence of age on infection dynamics within the susceptible and infectious populations, the susceptible class further subdivide into three distinct age groups: childhood $(S_{1})$, representing ages $0{-}14$ years; middle-aged $(S_{2})$, representing ages $15{-}59$ years; and senior $(S_{3})$, representing individuals over 60 years of age. The exposed, infected, and recovered classes remain consistent across these age groups. Furthermore, the model incorporates two key control strategies for TB in China. The first is the Directly Observed Treatment, Short-Course (DOTS) program, which enhances the recovery rate of infected individuals per year, represented by $(0<\psi <1)$. The second is the Bacillus Calmette-Guérin (BCG) immunization program, which provides immunity to newborns, denoted by $(0<\phi <1)$. The following system of ODEs defines the resulting model.

7 $$\begin{aligned} \begin{aligned}&\dfrac{dS_{1}}{dt} = \Lambda -m_{1} S_{1}-d_{1}S_{1}-\gamma _{1}S_{1}I, \\ &\dfrac{dS_{2}}{dt} =d_{1}S_{1}-m_{2}S_{2}-\gamma _{2}S_{2}I-d_{2}S_{2}, \\ &\dfrac{dS_{3}}{dt} = d_{2}S_{2}-m_{3}S_{3}-\gamma _{3}S_{3}I, \\ &\dfrac{dA}{dt} = (1-\beta )[(1-\phi )\gamma _{1}S_{1}I+\gamma _{2}S_{2}I+\gamma _{3}S_{3}I]- (\nu +d)A, \\ &\dfrac{dI}{dt}=\beta \big [(1-\phi )\gamma _{1}S_{1}I+\gamma _{2}S_{2}I+\gamma _{3}S_{3}I\big ] - \big [d+(1+\psi )r+\mu \big ]I+\nu A +\eta R, \\ &\dfrac{dR}{dt}= (1+\psi )rI-(d+\eta )R, \end{aligned} \end{aligned}$$

where, the parameters and their descriptions are provided in Table 1.

**Fig. 1:**
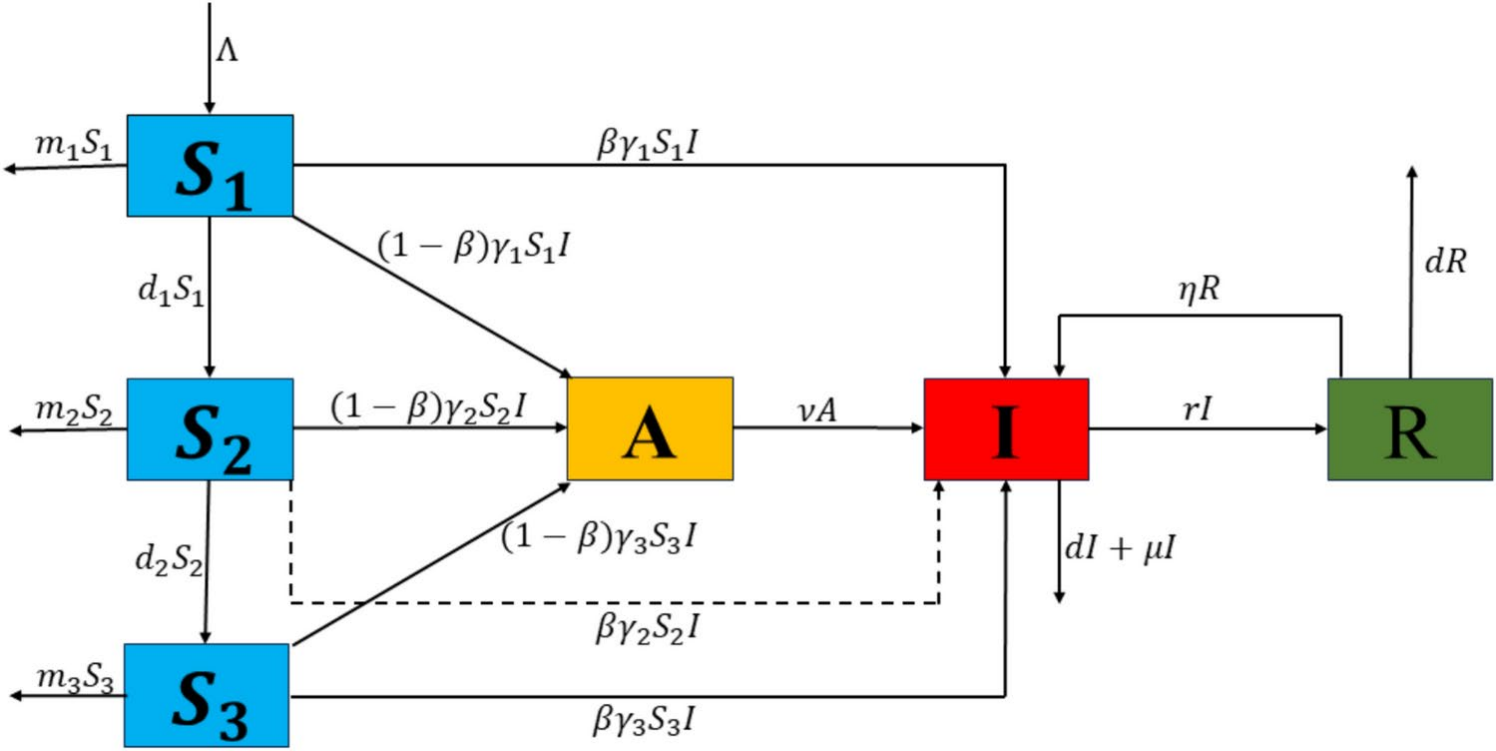
Compartment diagram of the TB model.

The above ODEs model (7) is further extended into a fractional-order system of order ($\varphi$) using the Caputo fractional derivative, as shown in Fig. 1. The motivation for employing the Caputo derivative lies in its ability to effectively capture memory effects and non-local behaviors, which are crucial for understanding the complex dynamics of TB infection. A key advantage of the Caputo definition is its compatibility with classical initial conditions, as its Laplace transform requires only integer-order derivatives of the initial values, making it more practical for real-world applications. Moreover, its initial conditions retain the same form as those in integer-order differential equations, enhancing its applicability in engineering and other applied sciences where such formulations have well-understood physical meanings. Additionally, the Caputo derivative avoids certain mathematical inconsistencies present in other fractional definitions, such as hyper-singular improper integrals, mass balance errors, and non-zero derivatives of constants, ensuring a more robust and physically meaningful representation of the system . These features make it a compelling choice for extending classical epidemiological models to fractional-order frameworks, allowing for improved accuracy in disease modeling while maintaining consistency with conventional differential equations.^36,37^ Moreover, in fractional-order systems, maintaining dimensional consistency is essential to ensure that both sides of the equations retain uniform units of measurement. To preserve this consistency, a common approach involves modifying the parameters on the right-hand side of the equations, typically by raising their power to ($\varphi$). Thus, the resulting system is represented as follows:

8 $$\begin{aligned} \begin{aligned}&^{C} _{0}{\mathfrak {D}}^{\varphi }_{t} S_{1} = \Lambda ^{\varphi } - m_{1}^{\varphi } S_{1}-d_{1}^{\varphi } S_{1}-\gamma ^{\varphi }_{1}S_{1}I, \\ &^{C} _{0}{\mathfrak {D}}^{\varphi }_{t} S_{2} = d^{\varphi }_{1}S_{1} - m^{\varphi }_{2}S_{2}-\gamma ^{\varphi }_{2}S_{2}I-d^{\varphi }_{2}S_{2}, \\ &^{C} _{0}{\mathfrak {D}}^{\varphi }_{t} S_{3}= d_{2}^{\varphi } S{2}-m_{3}^{\varphi } S_{3}-\gamma _{3}^{\varphi } S_{3}I, \\ &^{C} _{0}{\mathfrak {D}}^{\varphi }_{t} A = (1-\beta ^{\varphi })[(1-\phi ^{\varphi })\gamma _{1}^{\varphi } S_{1}I+\gamma _{2}^{\varphi } S_{2}I+\gamma _{3}^{\varphi } S_{3}I]- (\nu ^{\varphi }+d^{\varphi })A, \\ &^{C} _{0}{\mathfrak {D}}^{\varphi }_{t} I = \beta ^{\varphi } \big [(1-\phi ^{\varphi })\gamma _{1}^{\varphi } S_{1}I+\gamma _{2}^{\varphi } S_{2}I+\gamma _{3}^{\varphi } S_{3}I\big ] - \big [d^{\varphi }+(1+\psi ^{\varphi })r^{\varphi }+\mu ^{\varphi } \big ]I+\nu ^{\varphi } A +\eta ^{\varphi } R, \\ &^{C} _{0}{\mathfrak {D}}^{\varphi }_{t} R = (1+\psi ^{\varphi })r^{\varphi } I - (d^{\varphi }+\eta ^{\varphi })R, \end{aligned} \end{aligned}$$

subject to initial conditions:

9 $$\begin{aligned} S_{1}(0)=S_{1_0}, S_{2}(0)=S_{2_0}, S_{3}(0)=S_{3_0}, A(0)=A_{0}, I(0)=I_{0}, R(0)=R_{0}. \end{aligned}$$

**Table 1:** Parameter description.

Parameters	Description	Values	Sources
$\Lambda$	Annual birth rate	$1.623e+07$	^35^
*d*	Natural death rate	0.0067	^35^
$m_{1}$	Mortality rate of younger age group	0.0017	^35^
$m_{2}$	Mortality rate of middle age group	0.0023	^35^
$m_{3}$	Mortality rate of senior age group	0.0367	^35^
*r*	Recovery rate	0.496	^35^
$\beta$	Fraction of fast developing infection	0.05	^35^
$\nu$	Re-activation rate of latent TB	6	^35^
$\gamma _{1}$	Infection rate in younger age group	$1.182e-10$	Fitted
$\gamma _{2}$	Infection rate in middle age group	$5.265e-09$	Fitted
$\gamma _{3}$	Infection rate of senior age group	$2.575e-09$	Fitted
$d_{1}$	Rate of conversion from the susceptible children	0.0729	Fitted
	to middle aged category		
$d_{2}$	Rate of conversion from the susceptible middle	0.00618	Fitted
	aged to older group		
$\varphi$	Fractional order	0.93	Fitted
$\mu$	Disease induced death rate	0.0025	^35^
$\psi$	Annual recovery increment due to Directly Observed	0.51	^35^
	Treatment, Short-course (DOTS)		
$\phi$	Immunity rate of the Bacillus Calmette	0.9	^35^
	Guerin (BCG) vaccine		
$\eta$	Re-infection rate among successfully treated TB cases	0.00341	^35^

## Model analysis

### Non-negativity and boundedness

To ensure the biological feasibility of the model, we establish the non-negativity and boundedness of solutions. Before proceeding with the proof of theorem on the non-negativity of the model’s solutions, we first introduce the following lemma.^38^

#### Lemma 4.1

*Let a function* $\zeta \in C[c,d]$ *and the Caputo fractional derivative* $^{C} _{0}{\mathfrak {D}}^{\varphi }_{x} \zeta (x) \in C[c,d]$ *for* $0<\varphi \le 1,$ *then we have,*

$$\begin{aligned} \zeta (x) = \zeta (s) + \dfrac{1}{\Gamma (\varphi )}\, ^{C} _{0}{\mathfrak {D}}^{\varphi }_{x} \zeta (\tau )(x-s)^{\varphi }, \end{aligned}$$

*with* $$0\le \tau \le x, \forall \, x \in (c,d].$$

#### Remark 4.2

Consider a function $\zeta (x) \in C[0,d]$ and suppose that $^{C} _{0}{\mathfrak {D}}^{\varphi }_{x} \zeta (x) \in C[0,d]$ for $0<\varphi \le 1$. From Lemma1 it follows that if $^{C} _{0}{\mathfrak {D}}^{\varphi }_{x} \zeta (x) \ge 0$, for all $x \in (0,d]$ then $\zeta (x)$ is non-decreasing. Conversely, if $^{C} _{0}{\mathfrak {D}}^{\varphi }_{x} \zeta (x) \le 0$, for all $x \in (0,d]$, then $\zeta (x)$ is non-increasing for all $x \in (0,d]$.

#### Theorem 4.3

*All solutions of the system* (8) *are non-negative and are remains in with non-negative initial conditions remain positive for all* $t \ge 0$.

$$\begin{aligned} {\mathcal {R}}_{+}^{6}=\big \{{\mathcal {G}}: {\mathcal {G}}=(S_1 , S_2, S_3,A,I,R)\in {\mathcal {R}}^{6}, {\mathcal {G}}>0 \big \}. \end{aligned}$$

#### Proof

We will prove the non-negativity of solutions for our system (8) by using the Lemma 1. Since,

$$\begin{aligned}&^{C} _{0}{\mathfrak {D}}^{\varphi }_{t} S_{1} (t) \Big |_{S_{1}=0} = \Lambda ^{\varphi } \ge 0, \\ &^{C} _{0}{\mathfrak {D}}^{\varphi }_{t} S_{2} (t) \Big |_{S_{2}=0} = d_{1}^{\varphi }S_{1} \ge 0, \\ &^{C} _{0}{\mathfrak {D}}^{\varphi }_{t} S_{3} (t) \Big |_{S_{3}=0} = d_{2}^{\varphi }S_{2} \ge 0, \\ &^{C} _{0}{\mathfrak {D}}^{\varphi }_{t} A(t) \Big |_{A=0} = (1-\beta ^{\varphi })[(1-\phi ^{\varphi })\gamma _{1}^{\varphi } S_{1}I+\gamma _{2}^{\varphi } S_{2}I+\gamma _{3}^{\varphi } S_{3}I] \ge 0, \\ &^{C} _{0}{\mathfrak {D}}^{\varphi }_{t} I(t) \Big |_{I=0} = \nu ^{\varphi } A +\eta ^{\varphi } R \ge 0, \\ &^{C} _{0}{\mathfrak {D}}^{\varphi }_{t} R(t) \Big |_{R=0} = (1+\psi ^{\varphi })r^{\varphi } I \ge 0. \end{aligned}$$

As, a result $\forall t >0$, the solutions of the system remain positive and they will remain within ${\mathcal {R}}_{+}^{6}$. $\square$

#### Theorem 4.4

*The bounded region* ${\mathfrak {P}}=\biggl \{\big (S_{1},S_{2},S_{3},A,I,R\big ) \rightarrow {\mathbb {R}} _{+}^{6}: 0 \le {\mathcal {T}}(t) \le \dfrac{\Lambda }{{\hat{d}}}\biggr \}$ *serves as a positively invariant set for the system* (8)*, attracting all solutions that remain positive.*

#### Proof

Adding all equation of the system (8), we get

$$\begin{aligned} ^{C} _{0}{\mathfrak {D}}^{\varphi }_{t} {\mathcal {T}}(t)&= \Lambda ^{\varphi } -m_{1} ^{\varphi } S_{1}-m_{2}S_{2}-m_{3}^{\varphi } S_{3}-d^{\varphi } A-d^{\varphi } I-\mu ^{\varphi } I -d^{\varphi } R, \\ &= \Lambda ^{\varphi } - {\hat{d}} \bigl (S_{1}+S_{2}+S_{3}+A+I+R\bigr ) \\ &= \Lambda ^{\varphi } - {\hat{d}} {\mathcal {T}}, \end{aligned}$$

where, ${\hat{d}}= \min \bigl \{m_{1} ^{\varphi },m_{2}^{\varphi },m_{3} ^{\varphi },d^{\varphi },d^{\varphi }+\mu ^{\varphi }\bigr \}$. Applying the Laplace transform of Caputo derivative, we get

$$\begin{aligned}&s^{\varphi } {\mathcal {T}}(s) - {\mathcal {T}}(0) s^{\varphi -1} = \dfrac{\Lambda }{s} - {\hat{d}} {\mathcal {T}}(s), \\ &{\mathcal {T}}(s)= \dfrac{\Lambda }{s(s^{\varphi }+{\hat{d}})} + {\mathcal {T}}(0) \dfrac{s^{\varphi -1}}{s^{\varphi }+{\hat{d}}}, \\ &{\mathcal {T}}(t)= \dfrac{\Lambda }{{\hat{d}}} {\mathcal {L}}^{-1} \bigg [\dfrac{{\hat{d}}}{s(s^{\varphi }+{\hat{d}})}\bigg ] + {\mathcal {T}}(0) {\mathcal {L}}^{-1} \bigg [\dfrac{s^{\varphi -1}}{s^{\varphi }+{\hat{d}}}\bigg ], \\ &{\mathcal {T}}(t)= \dfrac{\Lambda }{{\hat{d}}} - \bigg [\dfrac{\Lambda }{{\hat{d}}} -{\mathcal {T}}(0)\bigg ] E_{a}(-{\hat{d}} t^{\varphi }). \end{aligned}$$

It follows that, $0 \le {\mathcal {T}}(t) \le \dfrac{\Lambda }{{\hat{d}}}$, as $t \rightarrow \infty$. Therefore, ${\mathcal {T}}(t)$ is bounded and all solutions begin in ${\mathfrak {P}}$ will remains in ${\mathfrak {P}}$. As a result, we obtained the following positive invariant set of model (8). This result ensures that the total population remains within a finite, biologically meaningful domain, reinforcing the model’s reliability. $\square$

### Existence and uniqueness of solution

This section provides a explicit proof of the existence and uniqueness of the solution to the fractional order TB model (8) using Banach contraction theorem.

#### Theorem 4.5

*Assuming the initial values of the model* (8) *are non-negative, the model* (8) *has a unique solution in* ${\mathbb {R}}^{6}_{+}$, *for all* $t \ge 0$.

#### Proof

Let the RHS of the fractional model (8) is written by

$$\begin{aligned}&f_{1} = \Lambda ^{\varphi } - m_{1}^{\varphi } S_{1}-d_{1}^{\varphi } S_{1}-\gamma ^{\varphi }_{1}S_{1}I, \\ &f_{2} = d^{\varphi }_{1}S_{1} - m^{\varphi }_{2}S_{2}-\gamma ^{\varphi }_{2}S_{2}I-d^{\varphi }_{2}S_{2}, \\ &f_{3}= d_{2}^{\varphi } S_{2}-m_{3}^{\varphi } S_{3}-\gamma _{3}^{\varphi } S_{3}I, \\ &f_{4}= (1-\beta ^{\varphi })[(1-\phi ^{\varphi })\gamma _{1}^{\varphi } S_{1}I+\gamma _{2}^{\varphi } S_{2}I+\gamma _{3}^{\varphi } S_{3}I]- (\nu ^{\varphi }+d^{\varphi })A, \\ &f_{5} = \beta ^{\varphi } \big [(1-\phi ^{\varphi })\gamma _{1}^{\varphi } S_{1}I+\gamma _{2}^{\varphi } S_{2}I+\gamma _{3}^{\varphi } S_{3}I\big ] - \big [d^{\varphi }+(1+\psi ^{\varphi })r^{\varphi }+\mu ^{\varphi } \big ]I+\nu ^{\varphi } A +\eta ^{\varphi } R, \\ &f_{6} = (1+\psi ^{\varphi })r^{\varphi } I - (d^{\varphi }+\eta ^{\varphi })R. \end{aligned}$$

Now, we find for every $S_{1}, \overline{S_{1}} \in {\mathbb {R}}_{+}^{6}$ that

10 $$\begin{aligned} \Vert f_{1}(t,S_{1})- f_{1}(t,\overline{S_{1}}) \Vert&= \Vert - (m_{1}^{\varphi }+d_{1}^{\varphi } + \gamma _{1}^{\varphi } I)S_{1} + (m_{1}^{\varphi }+d_{1}^{\varphi } + \gamma _{1}^{\varphi } I)\overline{S_{1}}\Vert , \nonumber \\&= \Vert - (m_{1}^{\varphi }+d_{1}^{\varphi } + \gamma _{1}^{\varphi } I) (S_{1}-\overline{S_{1}}) \Vert . \end{aligned}$$

Since, $S_{1}, S_{2}, S_{3}, A, I, R$ are bounded functions, i.e. $\Vert S_{1} \Vert \le k_{1},$ $\Vert S_{2} \Vert \le k_{2},$ $\Vert S_{3} \Vert \le k_{3},$ $\Vert A \Vert \le k_{4}, \Vert I \Vert \le k_{5}, \Vert R \Vert \le k_{6}$, by the property of norm, the Eq. (10) can be reformulated as,

11 $$\begin{aligned} \Vert f_{1}(t,S_{1})- f_{1}(t,\overline{S_{1}}) \Vert \le \varTheta _{1} \Vert S_{1}-\overline{S_{1}}\Vert , \end{aligned}$$

where, $\varTheta _{1} = m_{1}^{\varphi }+d_{1}^{\varphi } + \gamma _{1}^{\varphi } k_{5}$. Similarly, it can be demonstrated that

12 $$\begin{aligned}&\Vert f_{2}(t,S_{2})- f_{2}(t,\overline{S_{2}}) \Vert \le \varTheta _{2} \Vert S_{2}-\overline{S_{2}}\Vert , \end{aligned}$$

13 $$\begin{aligned}&\quad \Vert f_{3}(t,S_{3})- f_{3}(t,\overline{S_{3}}) \Vert \le \varTheta _{3} \Vert S_{3}-\overline{S_{3}}\Vert , \end{aligned}$$

14 $$\begin{aligned}&\quad \Vert f_{4}(t,A)- f_{4}(t,{\overline{A}}) \Vert \le \varTheta _{4} \Vert A-{\overline{A}}\Vert , \end{aligned}$$

15 $$\begin{aligned}&\quad \Vert f_{5}(t,I)- f_{6}(t,{\overline{I}}) \Vert \le \varTheta _{5} \Vert I-{\overline{I}}\Vert , \end{aligned}$$

16 $$\begin{aligned}&\quad \Vert f_{6}(t,R)- f_{6}(t,{\overline{R}}) \Vert \le \varTheta _{6} \Vert R-{\overline{R}}\Vert , \end{aligned}$$

where, $\varTheta _{2}= m_{2}^{\varphi }+\gamma _{2}^{\varphi }k_{5}+d_{2}^{\varphi },$ $\varTheta _{3}= m_{3}^{\varphi }+\gamma _{3}^{\varphi }k_{5},$ $\varTheta _{4}=\nu ^{\varphi }+d^{\varphi },$ $\varTheta _{5}= \beta ^{\varphi } \big [(1-\phi ^{\varphi })\gamma _{1}^{\varphi } S_{1}k_{5}+\gamma _{2}^{\varphi } S_{2}k_{5}+\gamma _{3}^{\varphi } S_{3}k_{5}\big ],$ $\varTheta _{6} = d^{\varphi }+\eta ^{\varphi }.$ It is evident that all kernels of $f_{i}$ contract and meet the Lipschitz condition if $0< \varTheta _{i}<1, i=1,\ldots ,6$.

By integrating both sides of the model (8), we obtain

$$\begin{aligned}&S_{1}(t)-S_{1}(0)= \dfrac{1}{\Gamma (\varphi )} \int _{0}^{t} f_{1}(s,S_{1}) (t-s)^{\varphi -1} ds, \\ &S_{2}(t)-S_{2}(0)= \dfrac{1}{\Gamma (\varphi )} \int _{0}^{t} f_{2}(s,S_{2}) (t-s)^{\varphi -1} ds, \\ &S_{3}(t)-S_{3}(0)= \dfrac{1}{\Gamma (\varphi )} \int _{0}^{t} f_{3}(s,s_{3}) (t-s)^{\varphi -1} ds, \\ &A(t)-A(0)= \dfrac{1}{\Gamma (\varphi )} \int _{0}^{t} f_{4}(s,A) (t-s)^{\varphi -1} ds, \\ &I(t)-I(0)= \dfrac{1}{\Gamma (\varphi )} \int _{0}^{t} f_{5}(s,I) (t-s)^{\varphi -1} ds, \\ &R(t)-R(0)= \dfrac{1}{\Gamma (\varphi )} \int _{0}^{t} f_{6}(s,R) (t-s)^{\varphi -1} ds. \end{aligned}$$

Hence, we obtain the iterative scheme as,

17 $$\begin{aligned} \begin{aligned}&S_{1n}= S_{1_0}+ \dfrac{1}{\Gamma (\varphi )} \int _{0}^{t} (t-s)^{\varphi -1} f_{1}(s,S_{1(n-1)}) ds, \\ &S_{2n}=S_{2_0}+\dfrac{1}{\Gamma (\varphi )} \int _{0}^{t} (t-s)^{\varphi -1} f_{2}(s,S_{2(n-1)}) ds, \\ &S_{3n}=S_{3_0}+ \dfrac{1}{\Gamma (\varphi )} \int _{0}^{t} (t-s)^{\varphi -1} f_{3}(s,S_{3(n-1)}) ds, \\ &A_{n}=A_{0}+ \dfrac{1}{\Gamma (\varphi )} \int _{0}^{t} (t-s)^{\varphi -1} f_{4}(s,A_{n-1}) ds, \\ &I_{n}=I_{0}+ \dfrac{1}{\Gamma (\varphi )} \int _{0}^{t} (t-s)^{\varphi -1} f_{5}(s,I_{n-1}) ds, \\ &R_{n}=R_{0}+\dfrac{1}{\Gamma (\varphi )} \int _{0}^{t} (t-s)^{\varphi -1} f_{6}(s,R_{n-1}) ds. \end{aligned} \end{aligned}$$

We now formulate a recursive expression based on Eq. (17) as follows:

18 $$\begin{aligned} \begin{aligned}&\varOmega _{1n}(t)=S_{1n}-S_{1(n-1)}= \dfrac{1}{\Gamma (\varphi )} \int _{0}^{t} (t-s)^{\varphi -1} \big (f_{1}(s,S_{1(n-1)})-f_{1}(s,S_{1(n-2)})\big ) ds, \\ &\varOmega _{2n}(t)=S_{2n}-S_{2(n-1)}= \dfrac{1}{\Gamma (\varphi )} \int _{0}^{t} (t-s)^{\varphi -1} \big (f_{2}(s,S_{2(n-1)})-f_{2}(s,S_{2(n-2)})\big ) ds, \\ &\varOmega _{3n}(t)=S_{3n}-S_{3(n-1)}= \dfrac{1}{\Gamma (\varphi )} \int _{0}^{t} (t-s)^{\varphi -1} \big (f_{3}(s,S_{3(n-1)})-f_{3}(s,S_{3(n-2)})\big ) ds, \\ &\varOmega _{4n}(t)=A_{n}-A_{n-1}= \dfrac{1}{\Gamma (\varphi )} \int _{0}^{t} (t-s)^{\varphi -1} \big (f_{4}(s,A_{n-1})-f_{4}(s,A_{n-2})\big ) ds, \\ &\varOmega _{5n}(t)=I_{n}-I_{n-1}= \dfrac{1}{\Gamma (\varphi )} \int _{0}^{t} (t-s)^{\varphi -1} \big (f_{5}(s,I_{n-1})-f_{5}(s,I_{n-2})\big ) ds, \\ &\varOmega _{6n}(t)=R_{n}-R_{n-1}= \dfrac{1}{\Gamma (\varphi )} \int _{0}^{t} (t-s)^{\varphi -1} \big (f_{6}(s,R_{n-1})-f_{6}(s,R_{n-2})\big ) ds. \end{aligned} \end{aligned}$$

The norm of the first Equation of (18) can be written as

$$\begin{aligned} \Vert \varOmega _{1n}(t) \Vert&= \Vert S_{1n}-S_{1(n-1)} \Vert = \bigg \Vert \dfrac{1}{\Gamma (\varphi )} \int _{0}^{t} (t-s)^{\varphi -1} \big (f_{1}(s,S_{1(n-1)})-f_{1}(s,S_{1(n-2)})\big ) ds \bigg \Vert , \\ &\le \dfrac{1}{\Gamma (\varphi )} \int _{0}^{t} (t-s)^{\varphi -1} \Vert \big (f_{1}(s,S_{1(n-1)})-f_{1}(s,S_{1(n-2)})\big ) \Vert ds, \\ &\le \dfrac{\varTheta _{1}}{\Gamma (\varphi )} \int _{0}^{t} (t-s)^{\varphi -1} \Vert S_{1(n-1)}(s)-S_{1(n-2)}(s)\Vert ds, \\ &= \dfrac{\varTheta _{1}}{\Gamma (\varphi )} \int _{0}^{t} (t-s)^{\varphi -1} \Vert \varOmega _{1(n-1)}(t) \Vert ds, \\ &= \bigg (\dfrac{\varTheta _{1} t}{\Gamma (\varphi +1)}\bigg )^{n} \Vert S(0) \Vert . \end{aligned}$$

Similarly, we have

$$\begin{aligned} \Vert \varOmega _{jn}(t) \Vert \le \bigg (\dfrac{\varTheta _{j} t}{\Gamma (\varphi +1)}\bigg )^{n} \,\Vert {\mathcal {Z}}_{j}(0) \Vert ; \forall j=2,3,\ldots , 6, \end{aligned}$$

where, $$\big ({\mathcal {Z}}_{1},{\mathcal {Z}}_{2}, {\mathcal {Z}}_{3}, {\mathcal {Z}}_{4}, {\mathcal {Z}}_{5}, {\mathcal {Z}}_{6} \big ) = \big (S_{1}, S_{2}, S_{3}, A, I, R\big )$$.

Assume that there is $t=T$, s.t. $\dfrac{\varTheta _{j} T}{\Gamma (\varphi +1)} < 1, \forall \, j=1,\ldots , 6$. As $n \rightarrow \infty$, it is evident that for each $j=1,\ldots ,6$, $\Vert \varOmega _{jn}(t) \Vert \rightarrow 0$. Consequently, model (8) has a solution.

We now prove the uniqueness of solutions with the same initial conditions. Assume that the model (8) has two distinct solutions corresponding to the same initial values, which we denoted as $\hat{S_{1}}, \hat{S_{2}}, \hat{S_{2}}, {\hat{A}}, {\hat{I}}, {\hat{R}}$. We obtain,

19 $$\begin{aligned} S_{1}-\hat{S_{1}}= \dfrac{1}{\Gamma (\varphi )} \int _{0}^{t} (t-s)^{\varphi -1} \big (f_{1}(s,S_{1})-f_{1}(s,\hat{S_{1}})\big ) ds. \end{aligned}$$

The norm of the above equation is

$$\begin{aligned} \Vert S_{1}-\hat{S_{1}} \Vert = \dfrac{1}{\Gamma (\varphi )} \int _{0}^{t} (t-s)^{\varphi -1} \Vert f_{1}(s,S_{1})-f_{1}(s,\hat{S_{1}})\Vert ds. \end{aligned}$$

Thus, by applying the Lipschitz condition, we derive:

20 $$\begin{aligned}&\Vert S_{1}-\hat{S_{1}} \Vert \le \dfrac{\varTheta _{1} T}{\Gamma (\varphi +1)} \Vert S_{1}-\hat{S_{1}} \Vert . \end{aligned}$$

21 $$\begin{aligned}&\quad \bigg (1- \dfrac{\varTheta _{1} T}{\Gamma (\varphi +1)}\bigg ) \Vert S_{1}-\hat{S_{1}} \Vert \le 0. \end{aligned}$$

If $\Vert S_{1}-\hat{S_{1}} \Vert =0$, then $S_{1}=\hat{S_{1}}$, satisfies the inequality (21). $S_{1}(t)$ is therefore unique. Similarly, we demonstrates the uniqueness of $S_{1}(t), S_{2}(t), S_{3}(t), A(t), I(t), \text {and}, R(t)$. $\square$

### Reproduction number

In a fully susceptible population, the basic reproduction number, which is commonly represented as ${\mathscr {R}}_{0}$, is the number of secondary cases produced by a single infectious individual. It serves as a key metric for assessing the potential for disease transmission within a community. If ${\mathscr {R}}_{0}<1$ the infection dies out over time, whereas if ${\mathscr {R}}_{0}>1$, the infection persists and continues to spread. In order to compute the reproduction number, we begin by identifying the disease-free equilibrium (DFE) point. The DFE corresponds to a state where the disease does not continue to exist in the population. It is obtained by setting

$$\begin{aligned} ^{C} _{0}{\mathfrak {D}}^{\varphi }_{t}S_{1}(t)=^{C} _{0}{\mathfrak {D}}^{\varphi }_{t}S_{2}(t)=^{C} _{0}{\mathfrak {D}}^{\varphi }_{t}S_{3}(t)=^{C} _{0}{\mathfrak {D}}^{\varphi }_{t}A(t)=^{C} _{0}{\mathfrak {D}}^{\varphi }_{t}I(t)=^{C} _{0}{\mathfrak {D}}^{\varphi }_{t}R(t)=0, \end{aligned}$$

and assuming that the infectious compartments in the model are zero. The DFE point for the model is obtained as

$$\begin{aligned} {\mathscr {E}}^{*}&=(S_{1}^{*},S_{2}^{*}.S_{3}^{*},0,0,0)\\&=\bigg (\dfrac{\Lambda ^{\varphi }}{(m_{1}^{\varphi }+d_{1}^{\varphi })},\dfrac{d_{1}^{\varphi } \Lambda ^{\varphi }}{(m_{1}^{\varphi }+d_{1}^{\varphi })(m_{2}^{\varphi }+d_{2}^{\varphi })}, \dfrac{d_{1}^{\varphi }d_{2}^{\varphi }\Lambda ^{\varphi }}{(m_{1}^{\varphi }+d_{1}^{\varphi })(m_{2}^{\varphi }+d_{2} ^{\varphi })d_{3}^{\varphi }},0,0,0\bigg ). \end{aligned}$$

Next, we utilize the next-generation matrix method^39^ to determine the basic reproduction number. Using this method, ${\mathscr {R}}_{0}$ is derived as,

22 $$\begin{aligned} {\mathscr {R}}_{0}=\dfrac{(\nu ^{\varphi }+\beta ^{\varphi } d^{\varphi })[(1-\phi ^{\varphi })\gamma _{1}^{\varphi }S_{1}^{*}+\gamma _{2}^{\varphi }S_{2}^{*}+\gamma _{3}^{\varphi }S_{3}^{*}]}{(\nu ^{\varphi }+d^{\varphi })[d^{\varphi }+(1+\psi ^{\varphi } )r^{\varphi }+\mu ^{\varphi }]}\bigg [1+\dfrac{\eta ^{\varphi }(1+\psi ^{\varphi })r^{\varphi }}{(d^{\varphi }+\eta ^{\varphi })(d^{\varphi }+(1+\psi ^{\varphi } )r^{\varphi }+\mu ^{\varphi })}\bigg ]. \end{aligned}$$

## Parameter estimation

The estimation of model parameters plays a fundamental role in validating epidemiological models. Accurate parameter estimation not only ensures the model reflects real-world dynamics but also enhances its predictive capabilities, aiding in the understanding of disease transmission and future epidemic trends. In this section, we estimate the model parameters by applying the least squares method, which minimizes the difference between the numerical solution for the infected population and the observed data of reported infections. To achieve this, we use MATLAB’s built-in function lsqcurvefit, which applies a non-linear least squares approach. The fractional-order model for TB incorporates 17 parameters. Of these, we estimate five key parameters $m_1,m_2,\gamma _{1},\gamma _{2},\gamma _{3}$, by fitting the model to TB case data from China spanning the years 2005 to 2016, as provided in.^35^ Additionally, we compute the root mean square error (RMSE) to compare the performance of the classical integer-order model with the fractional-order model. RMSE is calculated using the formula:

23 $$\begin{aligned} \text {RMSE}= \sqrt{\dfrac{1}{l}\sum _{k=0}^{l} \big (X(t_k)-X_{data}(t_k)\big )^{2}} \end{aligned}$$

Here, $X_{\text {data}}(t_k)$ and $X(t_k)$ represent the real and fitted data respectively at time $t_k$ and *l*, is the length of the corresponding data.

**Fig. 2:**
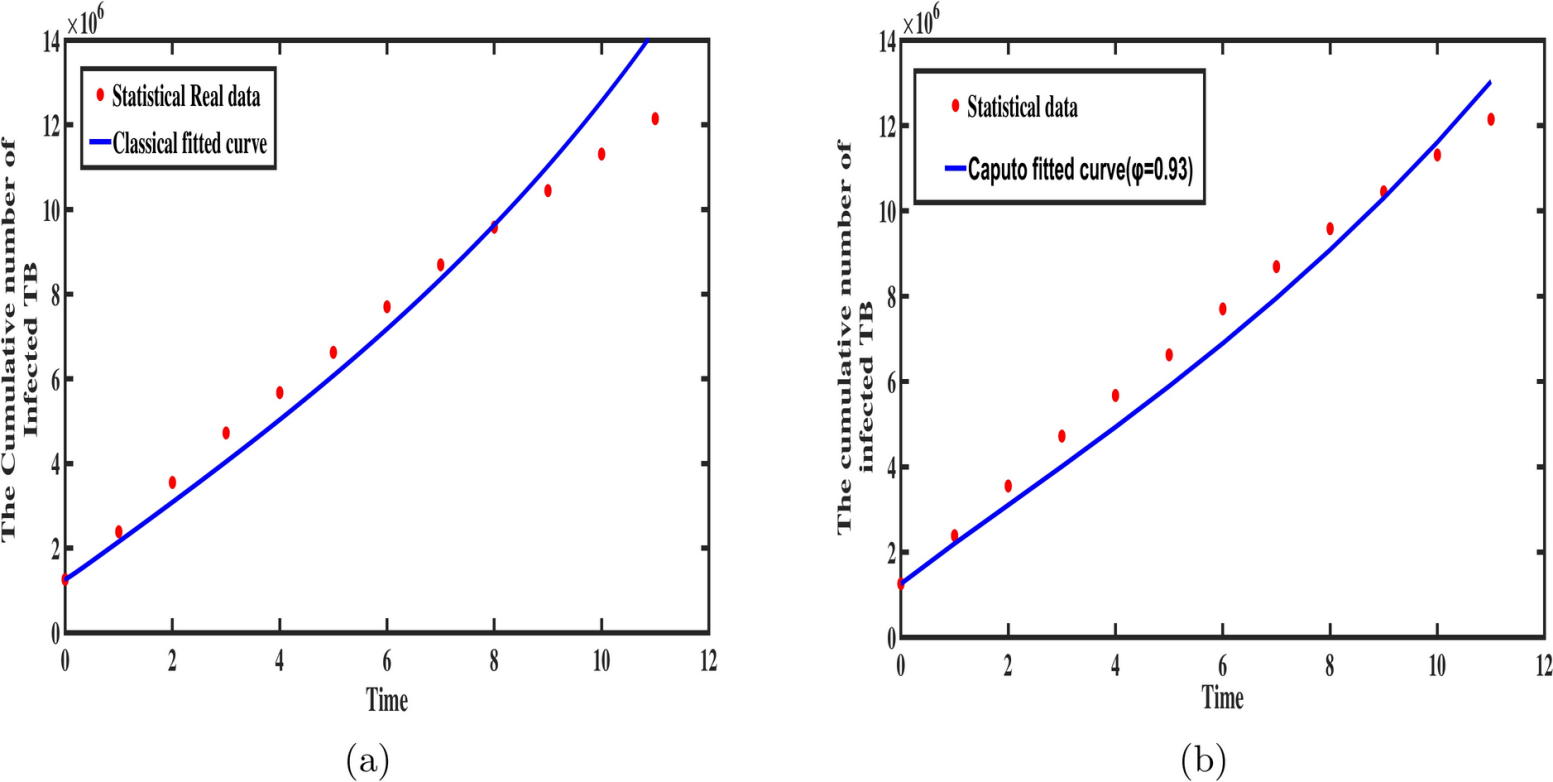
The behavior of classical and Caputo fractional models in comparison with statistical real cases.

In the case of fractional-order models, where the fractional derivative order, denoted by $\varphi$, can be adjusted to achieve the best fit between the model and observed data. This flexibility is a distinct advantage over deterministic integer-order models, where such adjustments are not possible. Let *Y*(*t*) represent the cumulative number of TB-infected people at time *t* in the model. The governing equation for this system is:

24 $$\begin{aligned} ^{C} _{0}{\mathfrak {D}}^{\varphi }_{t} Y(t) = \beta ^{\varphi } \big [(1-\phi ^{\varphi })\gamma _{1}^{\varphi } S_{1}I+\gamma _{2}^{\varphi } S_{2}I+\gamma _{3}^{\varphi } S_{3}I\big ] +\nu ^{\varphi } A +\eta ^{\varphi } R, \end{aligned}$$

where, I(t) represents the number of individuals in the infected compartment at time t, and $Z(t) = Y(t) - Y(t-1)$ corresponds to the newly infected TB cases at time t.

We estimated the optimal parameters of the fractional-order TB model by comparing the cumulative infected cases, with the model’s predictions. Our results indicate that the model produces the best fit to the observed data when $\varphi = 0.93$, demonstrating the presence of memory effects in TB transmission dynamics. The observed TB cases are depicted as solid red circles, while the best-fit curves of the model for both integer-order and fractional-order cases are shown in blue in Fig. 2a,b. The biological parameters used in the model, along with their best estimates obtained via the least squares method, are presented in Table 1. Table 2 compares the cumulative infected cases predicted by the classical integer-order model and the fractional-order model with the observed data, along with the RMSE values for both cases. The results show that the RMSE for the fractional order model is approximately $\epsilon = 5.89e+05$, while for the classical model, it is $\epsilon =8.24e+05$, representing a $28.6\%$ reduction in error in the fractional case. This demonstrates that the fractional-order model offers greater flexibility and improved accuracy in data fitting compared to the integer-order model. Based on these findings, we conclude that fractional-order models are better suited for capturing the dynamics of epidemics.

**Table 2:** Comparison of model predictions and RMSE for fractional order and classical order TB models.

Time(t)	Real data	Classical order predictions	Fractional order predication
			($$\varphi =0.93$$)
0	1.25e+06	1.25e+06	1.25e+06
1	2.38e+06	2.15e+06	2.2e+06
2	3.55e+06	3.08e+06	3.10e+06
3	4.72e+06	4.04e+06	4.00e+06
4	5.67e+06	5.03e+06	4.93e+06
5	6.62e+06	6.07e+06	5.89e+06
6	7.70e+06	7.17e+06	6.89e+06
7	8.69e+06	8.36e+06	7.95e+06
8	9.58e+06	9.63e+06	9.08e+06
9	1.04e+06	1.10e+06	1.03e+06
10	1.13e+06	1.25e+06	1.16e+06
11	1.21e+06	1.42e+06	1.30e+06
	RMSE($\epsilon$)	8.24e+05	5.89e+05

## Sensitivity analysis

In this section, we conduct a sensitivity analysis of the basic reproduction number ${\mathscr {R}}_{0}$. The objective of this analysis is to determine the influence of each model parameter on ${\mathscr {R}}_{0}$, quantified using the sensitivity index. The normalized forward sensitivity index of ${\mathscr {R}}_{0}$ with respect to a specific parameter ${\hat{p}}$ is given as:^40^

25 $$\begin{aligned} {\mathcal {I}}_{{\hat{p}}}= \dfrac{{\hat{p}}}{{\mathscr {R}}_{0}}\times \dfrac{\partial {\mathscr {R}}_{0}}{\partial {\hat{p}}}. \end{aligned}$$

A parameter’s impact on ${\mathscr {R}}_{0}$ is directly related to the absolute value of its sensitivity index, the larger the value, the greater the parameter’s influence. The sign of the index also provides insight into the direction of change: a positive index indicates that increasing the parameter enlarges ${\mathscr {R}}_{0}$, while a negative index suggests that an increase in the parameter reduces ${\mathscr {R}}_{0}$.

**Fig. 3:**
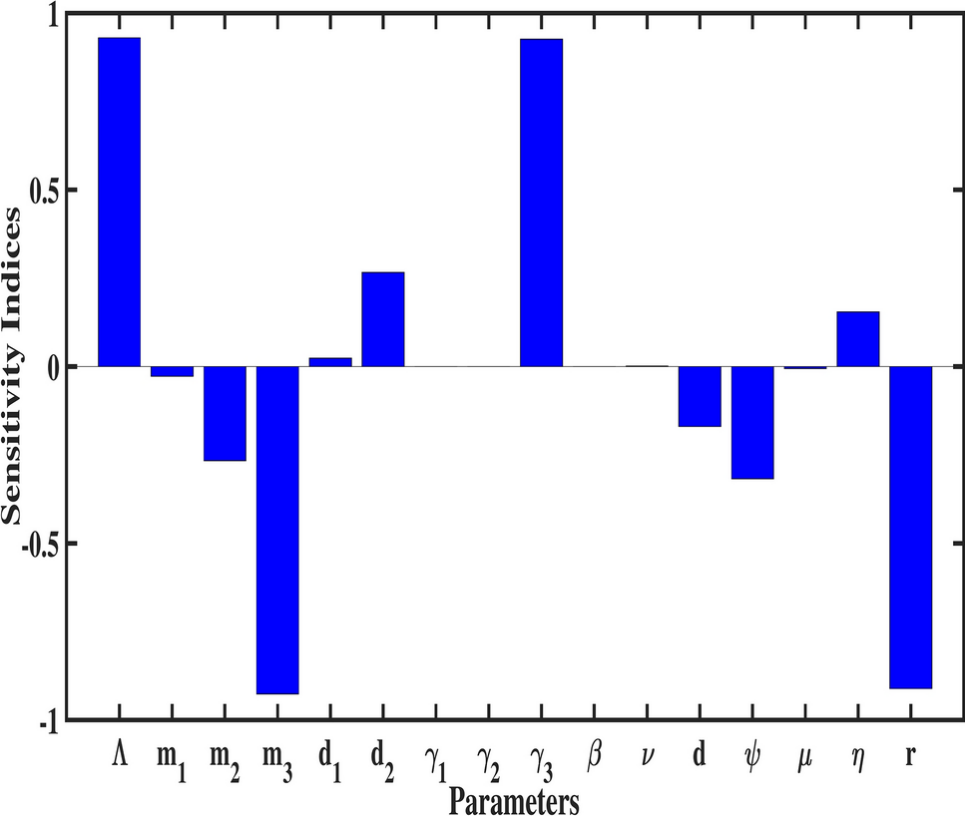
Bar plot showing the sensitivity indices of ${\mathscr {R}}_{0}$ for each parameter.

**Fig. 4:**
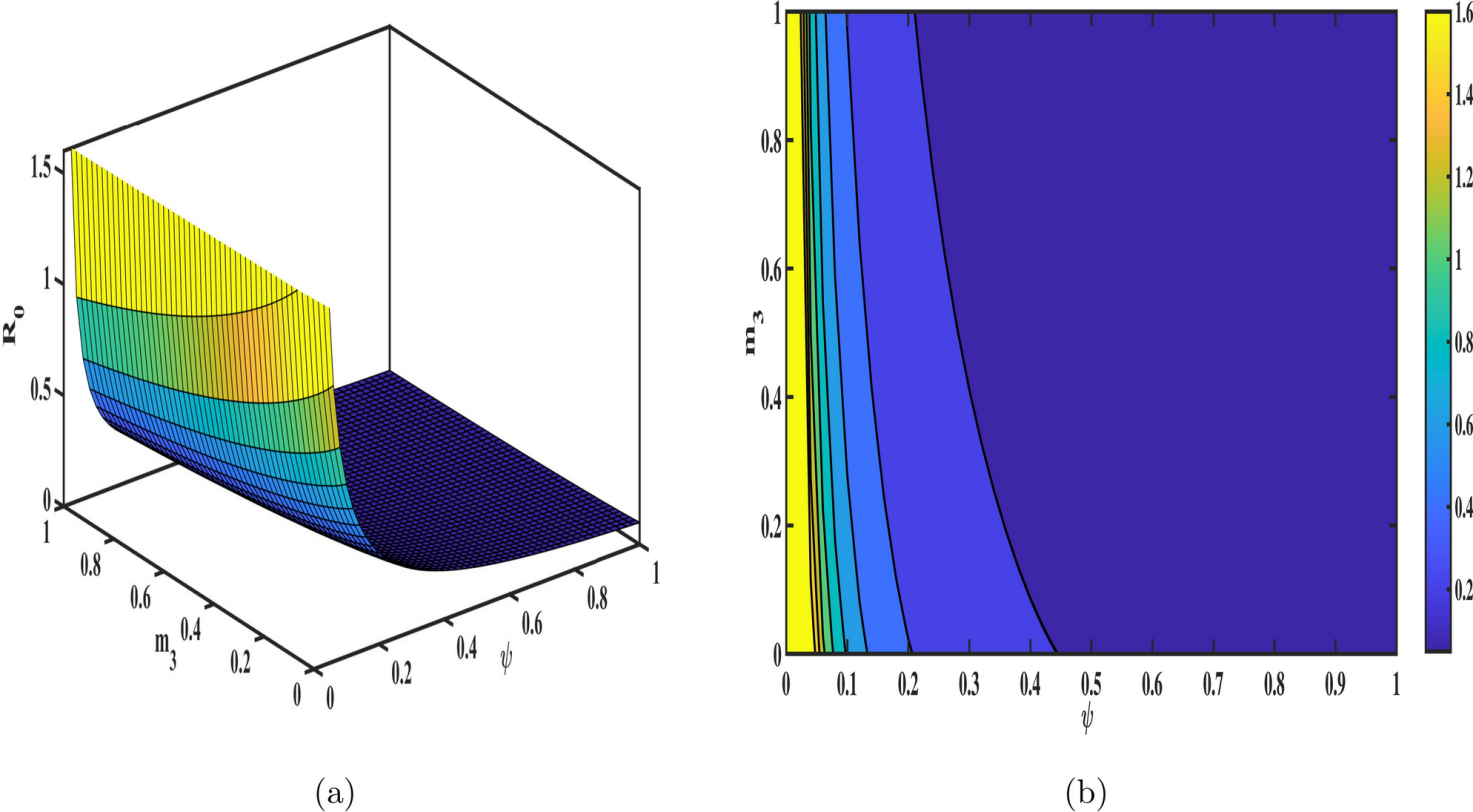
Influence of senior mortality rate ($m_{3}$) and annual recovery increment ($\psi$) on ${\mathscr {R}}_{0}$; (**a**) presents the 3D mesh plot, (**b**) shows the corresponding contour plot.

**Fig. 5:**
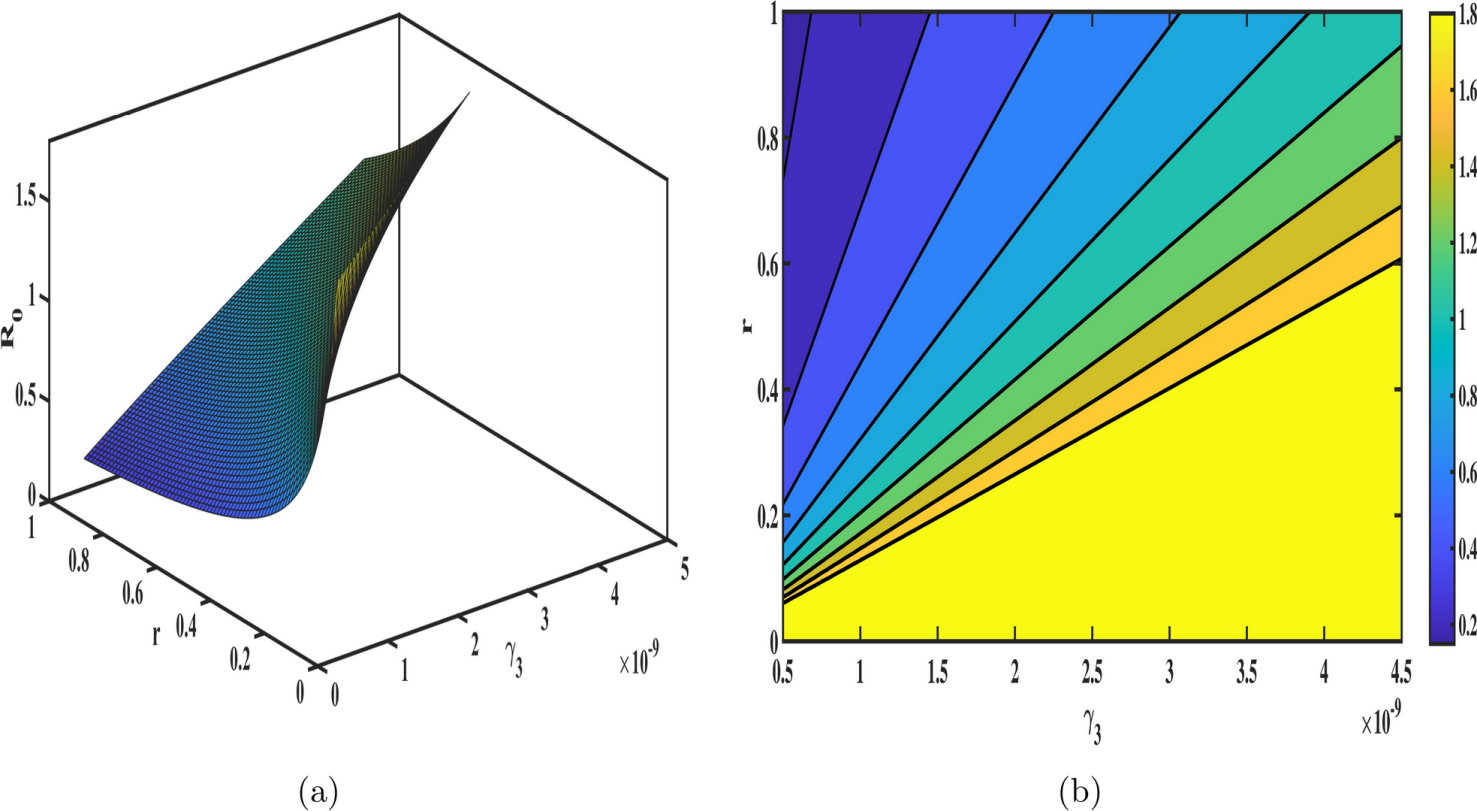
Influence of infection rate in seniors ($\gamma _{3}$) and recovery rate ($r$) on ${\mathscr {R}}_{0}$; (**a**) presents the 3D mesh plot, (**b**) shows the corresponding contour plot.

**Fig. 6:**
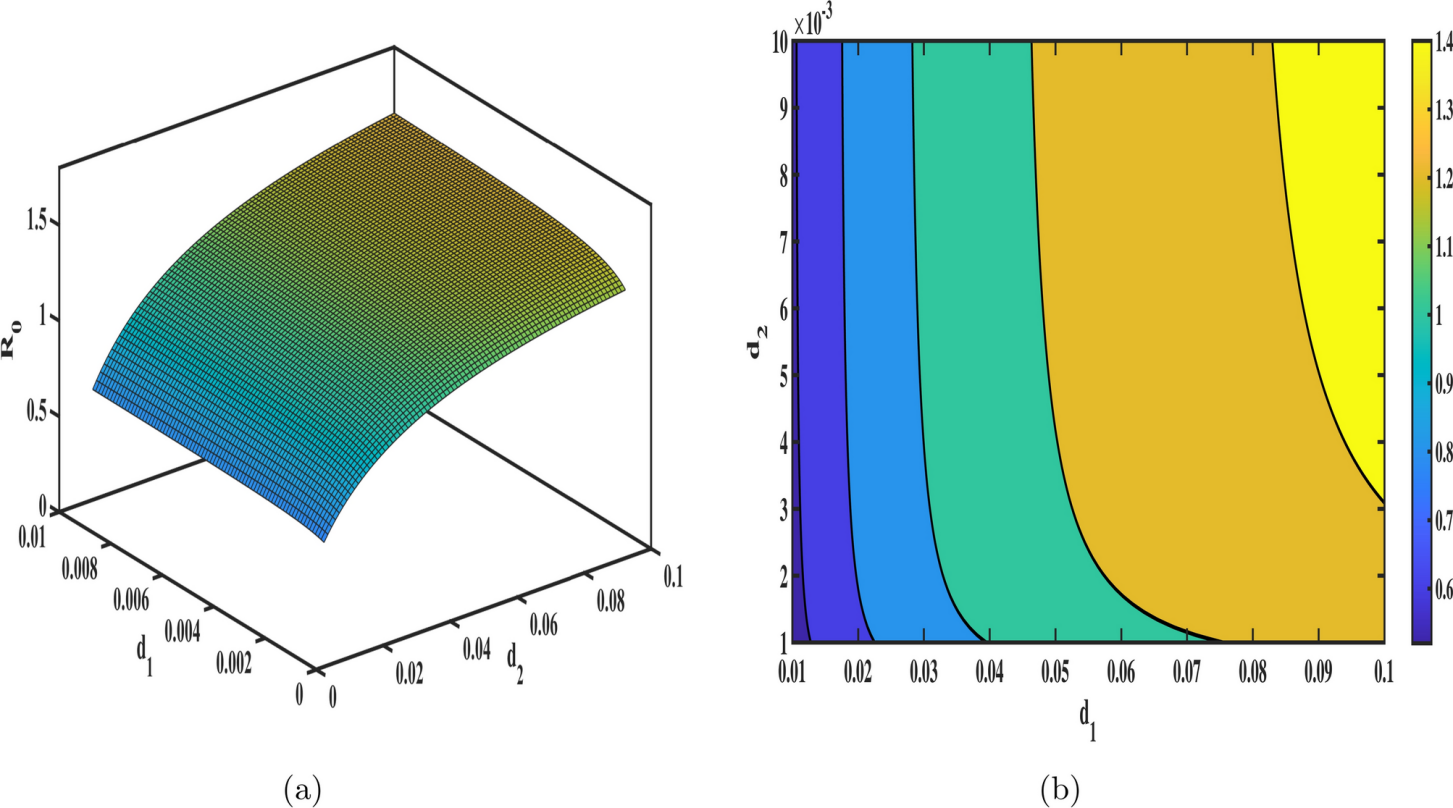
Combined impact of age transition rates ($d_{1}$) and ($d_{2}$) on ${\mathscr {R}}_{0}$; (**a**) presents the 3D mesh plot, (**b**) shows the corresponding contour plot.

**Fig. 7:**
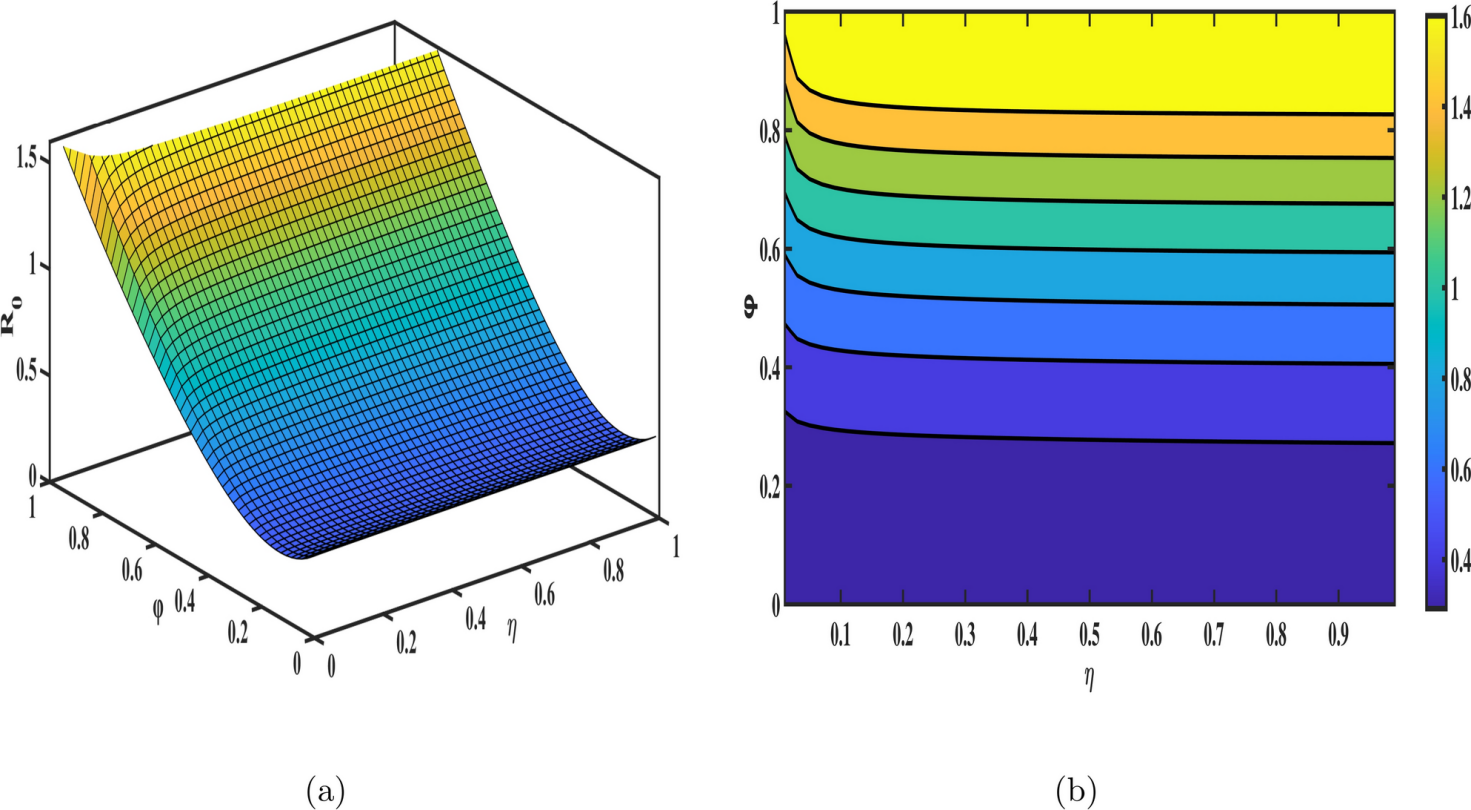
Variation of ${\mathscr {R}}_{0}$ with respect to the fractional order ($\phi$) and reinfection rate ($\eta$); (**a**) presents the 3D mesh plot, (**b**) shows the corresponding contour plot.

The sensitivity index for each parameter is calculated by substituting the parameter values from Table 1 into the sensitivity formula. The results are presented in Fig. 3, and the numerical values of the sensitivity indices are summarized in Table 3. Among the parameters, $\Lambda , m_3, \gamma _3,\, and \, r$ exhibit the most significant influence on ${\mathscr {R}}_{0}$, with sensitivity indices of $0.93,-0.961,0.9261, \text {and} \, -0.9108$ respectively. These findings underscore the critical role of the senior population in the dynamics of tuberculosis transmission. Specifically, reducing the infection rate ($\gamma _3$) through enhanced diagnostics, prophylaxis, and isolation measures, as well as improving the recovery rate (*r*) via effective treatment programs, can significantly mitigate the spread of TB. Furthermore, healthcare improvements tailored to seniors, such as regular screenings and latent TB management, are vital to controlling transmission. To provide a more comprehensive understanding, 3D surface meshes and contour plots are included to illustrate the impact of various parameters on ${\mathscr {R}}_{0}$ in Figs. 4, 5, 6 and 7. These visualization techniques are widely used in epidemiological modeling as they provide a clear representation of parameter interactions and their nonlinear effects on disease dynamics. Figure 4 demonstrates the combined effects of the annual recovery increment due to DOTS ($\psi$) and the senior population mortality rate ($m_{3}$) on ${\mathscr {R}}_{0}$. It emphasizes the crucial role of simultaneously raising $\psi$ and decreasing $m_{3}$ through improved healthcare measures to effectively control TB transmission. The Fig. 5 displays the variation with the recovery rate (*r*) and the infection rate in seniors ($\gamma _{3}$). A decrease in ($\gamma _{3}$) further reduces disease transmission, while a higher recovery rate significantly lowers ${\mathscr {R}}_{0}.$ The interaction between age group transition rates $d_{1},d_{2}$ and their effect on ${\mathscr {R}}_{0}$ is depicted in the Fig. 6. Additionally, the Fig. 7 illustrates how ${\mathscr {R}}_{0}$ is influenced by the fractional order ($\varphi$) and the reinfection rate ($\eta$). These results demonstrate how fractional dynamics interact with key epidemiological parameters to shape the transmission and control of TB.

**Table 3:** The values of sensitivity index of ${\mathscr {R}}_{0}$.

Parameters	Sensitivity indices
$\Lambda$	0.93
$m_{1}$	$-0.0276919$
$m_{2}$	$-0.266303$
$m_{3}$	$-0.926139$
$\gamma _{1}$	$3.774e-05$
$\gamma _{2}$	0.00016811
$\gamma _{3}$	0.926139
$d_{1}$	0.023993
$d_{2}$	0.266471
*d*	$-0.169845$
$\psi$	$-0.317324$
$\eta$	0.15462
*r*	$-0.910881$
$\beta$	0.000103
$\nu$	0.001565
$\mu$	$-0.00054605$

## Numerical simulation and discussion

In this section, we simulate the proposed TB disease model (8) governed by the Caputo fractional operator, which is autonomous in nature. The state variables $S_{1}(t), S_{2}(t), S_{3}(t), A(t), I(t)$ $\text {and},\, R(t)$ are evaluated under different biological parameter values (both fixed and fitted) and the optimized fractional-order parameter, obtained through the non-linear least squares curve-fitting approach. The simulations are performed using the fractional Adams method, a widely used numerical method that employs a predictor-corrector scheme, as detailed in.^41,42^ This method combines explicit and implicit procedures via the Adams-Bashforth-Moulton (ABM) fractional numerical technique to efficiently and accurately solve fractional-order differential equations. For the simulations, the time step size is chosen as $h=1e-02$, corresponding to the interval of time [0, 80]. The initial conditions of the system are specified as:^35^ $S_{1}(0)=26,504e+04, S_{2}(0)=94,197e+04, S_{3}(0)= 10,055 e+04, A(0)=1,174,03, I(0)= 1,259,308,\, \text {and} \,R(0)= 776,223$, with biological parameter values taken from Table 1. To demonstrate the method, the fractional differential equations are formulated in the following form:

26 $$\begin{aligned}&^{C} _{0}{\mathfrak {D}}^{\varphi }_{t} \vartheta (t) = {\mathcal {F}}(t,\vartheta (t)), \end{aligned}$$

27 $$\begin{aligned}&\vartheta ^{i}(0)=\vartheta _{0}^{i}; \, i=0,1,\ldots ,n-1, \end{aligned}$$

where, $\varphi >0, \text {and} \,\, i= \lceil \varphi \rceil$. The fractional differential equation stated above can be transformed into an equivalent Volterra integral equation, which is expressed as:

28 $$\begin{aligned} \vartheta (t)=\sum _{i=0}^{n-1} \vartheta _{0}^{i} \dfrac{t^{i}}{i!}+ \dfrac{1}{\Gamma (\varphi )} \int _{0}^{t} (t-\tau )^{\varphi -1} {\mathcal {F}}(\tau ,\vartheta (\tau )) d\tau . \end{aligned}$$

When applying Adams Bashforth Moulton’s generalized predictor-corrector approach, the uniform grid points are set to $t_k = kh, k=0,1,\ldots ,m \,\text {with}\, m \in {\mathbb {N}}$. Then, the following discretization of Eq. (28) is possible:

29 $$\begin{aligned} \vartheta _{k+1}&= \sum _{i=0}^{n-1} \vartheta _{0}^{i}\, \dfrac{t^{i}_{k+1}}{i!} + \dfrac{h^{\varphi }}{\Gamma (\varphi +2)} \bigg [\sum _{i=0}^{k}a_{i,k+1}\, {\mathcal {F}}(t_{i},\vartheta _{i}) + a_{k+1,k+1} {\mathcal {F}}(t_{k+1},\vartheta _{k+1}^{P})\bigg ], \end{aligned}$$

30 $$\begin{aligned} \vartheta _{k+1}^{P}&= \sum _{i=0}^{n-1} \vartheta _{0}^{i}\, \dfrac{t^{i}_{k+1}}{i!} + \dfrac{h^{\varphi }}{\Gamma (\varphi +2)} \bigg [\sum _{i=0}^{k}b_{i,k+1}\, {\mathcal {F}}(t_{i},\vartheta _{i})\bigg ], \end{aligned}$$

where, the weights are defined as:

31 $$\begin{aligned}&a_{i,k+1}= {\left\{ \begin{array}{l} k^{\varphi +1}-(k-\varphi )(k+\varphi )^{\varphi }; i=0 \\ (k-i+2)^{\varphi +1}+(k-i)^{\varphi +1}-2(k-i+1)^{\varphi +1}; 1\le i\le k \\ 1; i=k+1 \end{array}\right. } \end{aligned}$$

32 $$\begin{aligned}&\quad \text {and}\,\,\,\, b_{i,k+1}= (k-i+1)^{\varphi }-(k-i)^{\varphi }; \, \, i=0,1,\ldots ,k. \end{aligned}$$

The fractional Adams method, as discussed earlier, is used to simulate the Caputo model (8). The following is the system’s reduction to the corrector formula:

33 $$\begin{aligned} S_{1}(t_{n+1})= & S_{1}(t_{0}) + \dfrac{h^{\varphi }}{\Gamma (\varphi +2)} \sum _{i=0}^{k}a_{i,n+1}\big (\Lambda ^{\varphi } - m_{1}^{\varphi } S_{1}(t_{i})-d_{1}^{\varphi } S_{1}(t_{i})-\gamma ^{\varphi }_{1}S_{1}(t_{i})I(t_{i})\big ) \nonumber \\ & + \dfrac{h^{\varphi }}{\Gamma (\varphi +2)}\big (\Lambda ^{\varphi } - m_{1}^{\varphi } S_{1}^{P}(t_{n+1})-d_{1}^{\varphi } S_{1}^{P}(t_{n+1})-\gamma ^{\varphi }_{1}S_{1}^{P}(t_{n+1})I^{P}(t_{n+1})\big ), \end{aligned}$$

34 $$\begin{aligned} S_{2}(t_{n+1})= & S_{2}(t_{0}) + \dfrac{h^{\varphi }}{\Gamma (\varphi +2)} \sum _{i=0}^{k}a_{i,n+1} \big (d^{\varphi }_{1}S_{1}(t_{i}) - m^{\varphi }_{2}S_{2}(t_{i})-\gamma ^{\varphi }_{2}S_{2}(t_{i})I(t_{i})-d^{\varphi }_{2}S_{2}(t_{i})\big )\nonumber \\ & + \dfrac{h^{\varphi }}{\Gamma (\varphi +2)} \big (d^{\varphi }_{1}S_{1}^{P}(t_{n+1}) - m^{\varphi }_{2}S_{2}^{P}(t_{n+1})-\gamma ^{\varphi }_{2}S_{2}^{P}(t_{n+1})I^{P}(t_{n+1})-d^{\varphi }_{2} S_{2}^{P}(t_{n+1})\big ), \end{aligned}$$

35 $$\begin{aligned} S_{3}(t_{n+1})= & S_{3}(t_{0}) + \dfrac{h^{\varphi }}{\Gamma (\varphi +2)} \sum _{i=0}^{k}a_{i,n+1} \big (d_{2}^{\varphi } S_{2}(t_{i})-m_{3}^{\varphi } S_{3}(t_{i})-\gamma _{3}^{\varphi } S_{3}(t_{i})I(t_{i})\big ) \nonumber \\ & + \dfrac{h^{\varphi }}{\Gamma (\varphi +2)} \big (d_{2}^{\varphi } S_{2}^{P}(t_{n+1})-m_{3}^{\varphi } S_{3}^{P}(t_{n+1})-\gamma _{3}^{\varphi } S_{3}^{P}(t_{n+1})I^{P}(t_{n+1})\big ), \end{aligned}$$

36 $$\begin{aligned} A(t_{n+1})= & A(t_{0}) + \dfrac{h^{\varphi }}{\Gamma (\varphi +2)} \sum _{i=0}^{k}a_{i,n+1} \big ((1-\beta ^{\varphi })[(1-\phi ^{\varphi })\gamma _{1}^{\varphi } S_{1}(t_{i})I(t_{i})+\gamma _{2}^{\varphi } S_{2}(t_{i})I(t_{i}) \nonumber \\ & +\gamma _{3}^{\varphi } S_{3}(t_{i})I(t_{i})] - (\nu ^{\varphi }+d^{\varphi })A(t_{i})\big ) + \dfrac{h^{\varphi }}{\Gamma (\varphi +2)} \big ((1-\beta ^{\varphi })[(1-\phi ^{\varphi })\gamma _{1}^{\varphi } S_{1}^{P}(t_{n+1})I^{P}(t_{n+1}) \nonumber \\ & +\gamma _{2}^{\varphi } S_{2}^{P}(t_{n+1})I^{P}(t_{n+1})+\gamma _{3}^{\varphi } S_{3}^{P}(t_{n+1})I^{P}(t_{n+1})]- (\nu ^{\varphi }+d^{\varphi })A^{P}(t_{n+1})\big ), \end{aligned}$$

37 $$\begin{aligned} I(t_{n+1})= & I(t_{0}) + \dfrac{h^{\varphi }}{\Gamma (\varphi +2)} \sum _{i=0}^{k}a_{i,n+1} \big (\beta ^{\varphi } \big [(1-\phi ^{\varphi })\gamma _{1}^{\varphi } S_{1}(t_{i})I(t_{i})+\gamma _{2}^{\varphi } S_{2}(t_{i})I(t_{i})+\gamma _{3}^{\varphi } S_{3}(t_{i})I(t_{i})\big ]\nonumber \\ & - \big [d^{\varphi }+(1+\psi ^{\varphi })r^{\varphi }+\mu ^{\varphi } \big ]I(t_{i})+\nu ^{\varphi } A(t_{i}) +\eta ^{\varphi } R(t_{i})\big )+ \dfrac{h^{\varphi }}{\Gamma (\varphi +2)} \nonumber \\ & \times \big (\beta ^{\varphi } \big [(1-\phi ^{\varphi })\gamma _{1}^{\varphi } S_{1}^{P}(t_{n+1})I^{P}(t_{n+1}) +\gamma _{2}^{\varphi } S_{2}^{P}(t_{n+1})I^{P}(t_{n+1})+\gamma _{3}^{\varphi } S_{3}^{P}(t_{n+1})I^{P}(t_{n+1})\big ] \nonumber \\ & - \big [d^{\varphi }+(1+\psi ^{\varphi })r^{\varphi }+\mu ^{\varphi } \big ]I^{P}(t_{n+1})+\nu ^{\varphi } A^{P}(t_{n+1}) +\eta ^{\varphi } R^{P}(t_{n+1})\big ),\end{aligned}$$

38 $$\begin{aligned} R(t_{n+1})= & R(t_{0}) + \dfrac{h^{\varphi }}{\Gamma (\varphi +2)} \sum _{i=0}^{k}a_{i,n+1} \big ((1+\psi ^{\varphi })r^{\varphi } I(t_{i}) -(d^{\varphi }+\eta ^{\varphi })R(t_{i})\big ) \nonumber \\ & + \dfrac{h^{\varphi }}{\Gamma (\varphi +2)} \big ((1+\psi ^{\varphi })r^{\varphi } I^{P}(t_{n+1}) -(d^{\varphi }+\eta ^{\varphi })R^{P}(t_{n+1})\big ). \end{aligned}$$

and the predictor formula is given by,

39 $$\begin{aligned} S^{P}_{1}(t_{n+1})&= S_{1}(t_{0}) + \dfrac{h^{\varphi }}{\Gamma (\varphi +2)} \sum _{i=0}^{k}b_{i,n+1} \big (\Lambda ^{\varphi } - m_{1}^{\varphi } S_{1}(t_{i})-d_{1}^{\varphi } S_{1}(t_{i})-\gamma ^{\varphi }_{1}S_{1}(t_{i})I(t_{i})\big ), \end{aligned}$$

40 $$\begin{aligned} S_{2}^{P}(t_{n+1})&= S_{2}(t_{0}) + \dfrac{h^{\varphi }}{\Gamma (\varphi +2)} \sum _{i=0}^{k}b_{i,n+1} \bigg (d^{\varphi }_{1}S_{1}(t_{i}) - m^{\varphi }_{2}S_{2}(t_{i})-\gamma ^{\varphi }_{2}S_{2}(t_{i})I(t_{i})-d^{\varphi }_{2}S_{2}(t_{i})\bigg ), \end{aligned}$$

41 $$\begin{aligned} S_{3}^{P}(t_{n+1})&= S_{3}(t_{0}) + \dfrac{h^{\varphi }}{\Gamma (\varphi +2)} \sum _{i=0}^{k}b_{i,n+1} \big (d_{2}^{\varphi } S_{2}(t_{i})-m_{3}^{\varphi } S_{3}(t_{i})-\gamma _{3}^{\varphi } S_{3}(t_{i})I(t_{i})\big ), \end{aligned}$$

42 $$\begin{aligned} A^{P}(t_{n+1})&= A(t_{0}) + \dfrac{h^{\varphi }}{\Gamma (\varphi +2)} \sum _{i=0}^{k}b_{i,n+1} \big ((1-\beta ^{\varphi })[(1-\phi ^{\varphi })\gamma _{1}^{\varphi } S_{1}(t_{i})I(t_{i})+\gamma _{2}^{\varphi } S_{2}(t_{i})I(t_{i}) \nonumber \\&\quad +\gamma _{3}^{\varphi } S_{3}(t_{i})I(t_{i})]- (\nu ^{\varphi }+d^{\varphi })A(t_{i})\big ), \end{aligned}$$

43 $$\begin{aligned} I^{P}(t_{n+1})&= I(t_{0}) + \dfrac{h^{\varphi }}{\Gamma (\varphi +2)} \sum _{i=0}^{k}b_{i,n+1} \big (\beta ^{\varphi } \big [(1-\phi ^{\varphi })\gamma _{1}^{\varphi } S_{1}(t_{i})I(t_{i})+\gamma _{2}^{\varphi } S_{2}(t_{i})I(t_{i})+\gamma _{3}^{\varphi } S_{3}(t_{i})I(t_{i})\big ] \nonumber \\&\quad - \big [d^{\varphi }+(1+\psi ^{\varphi })r^{\varphi }+\mu ^{\varphi } \big ]I(t_{i})+\nu ^{\varphi } A(t_{i}) +\eta ^{\varphi } R(t_{i})\big ), \end{aligned}$$

44 $$\begin{aligned} R^{P}(t_{n+1})&= R(t_{0}) + \dfrac{h^{\varphi }}{\Gamma (\varphi +2)} \sum _{i=0}^{k}b_{i,n+1} \big ((1+\psi ^{\varphi })r^{\varphi } I(t_{i}) -(d^{\varphi }+\eta ^{\varphi })R(t_{i})\big ). \end{aligned}$$

**Fig. 8:**
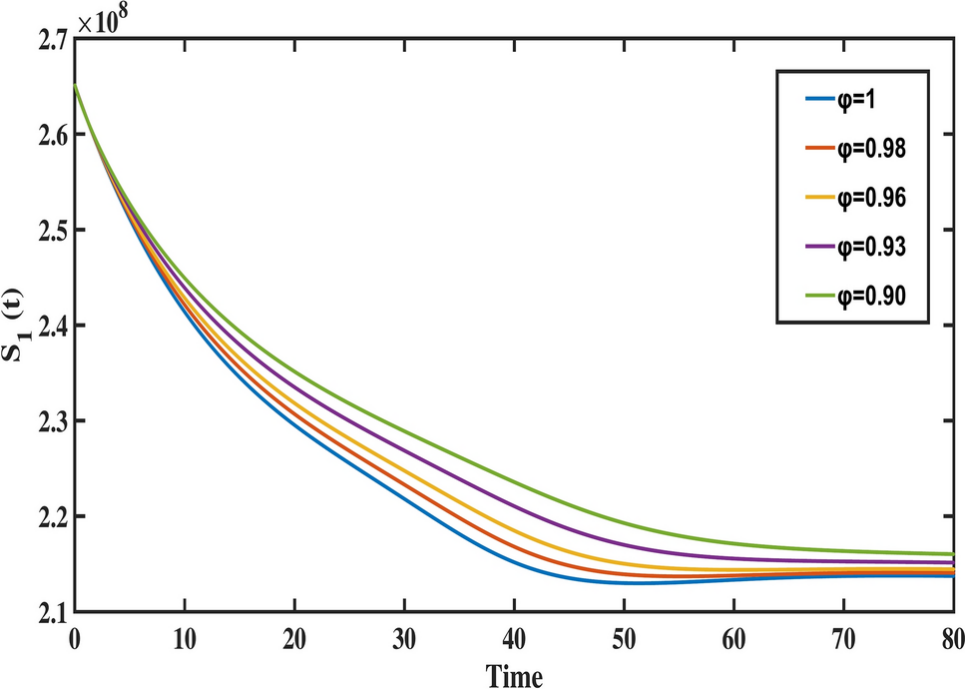
The behavior of child age susceptible population for different values of fractional order ($\varphi$).

**Fig. 9:**
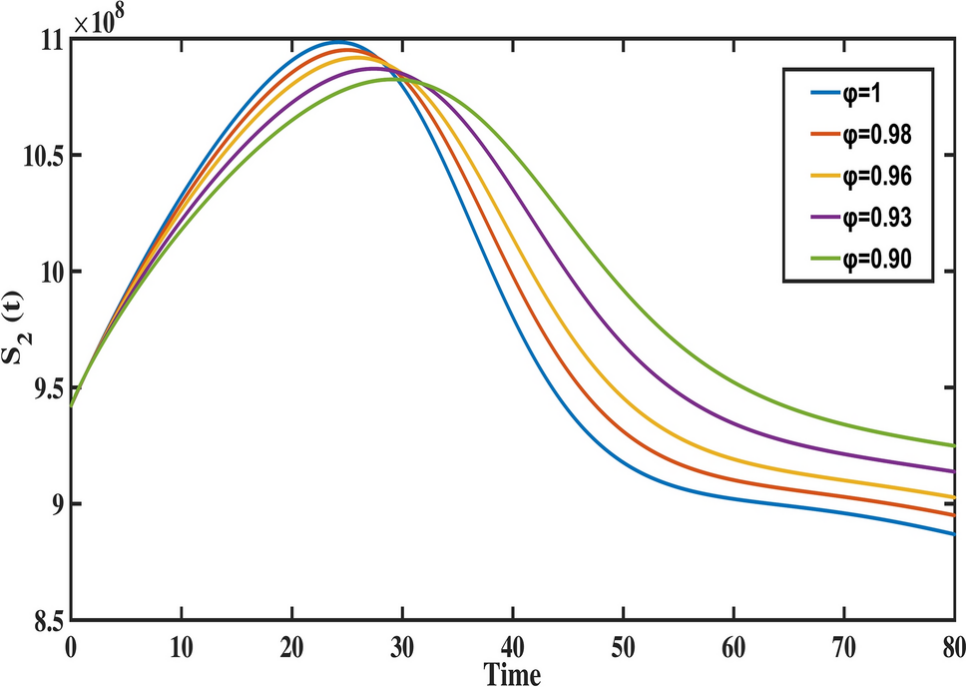
The behavior of middle aged susceptible population for different values of fractional order ($\varphi$).

**Fig. 10:**
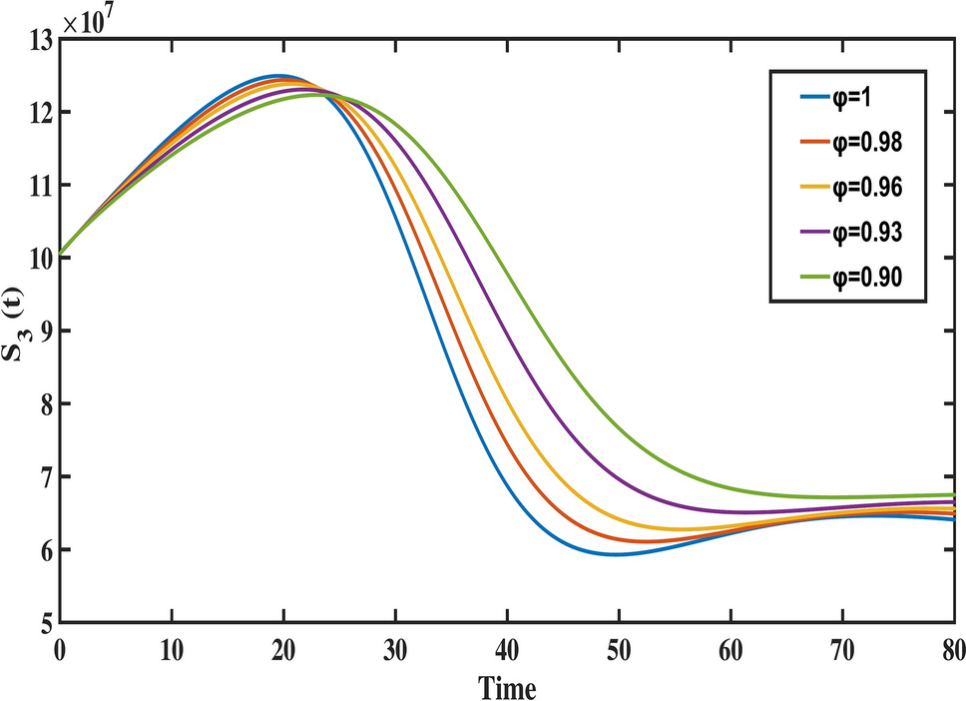
The behavior of older aged susceptible population for different values of fractional order ($\varphi$).

**Fig. 11:**
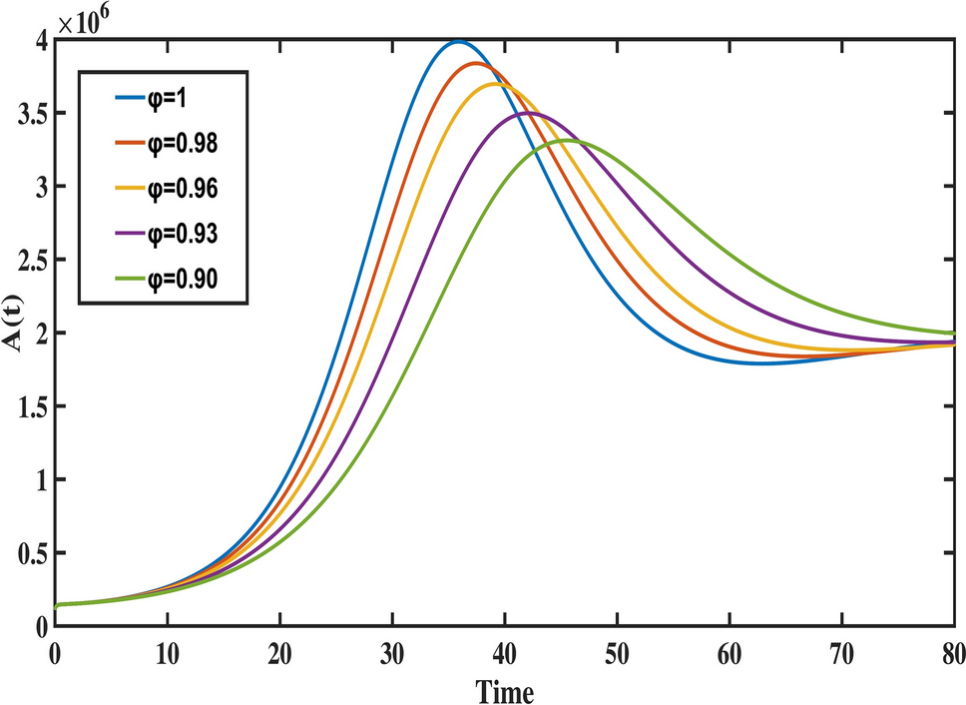
The behavior of exposed population for different values of fractional order ($\varphi$).

**Fig. 12:**
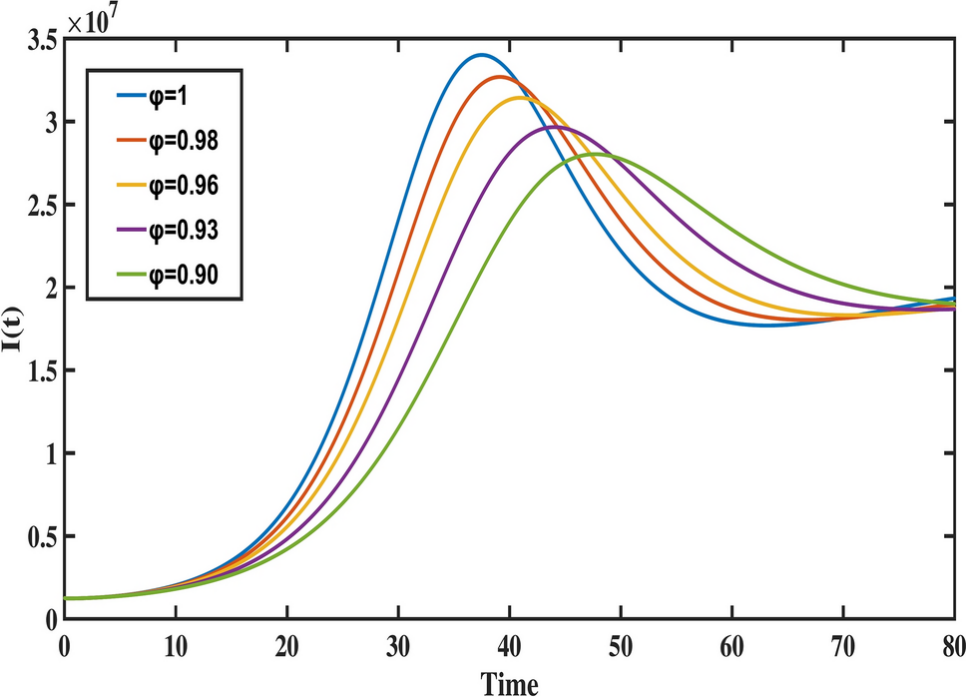
The behavior of infected population for different values of fractional order ($\varphi$).

**Fig. 13:**
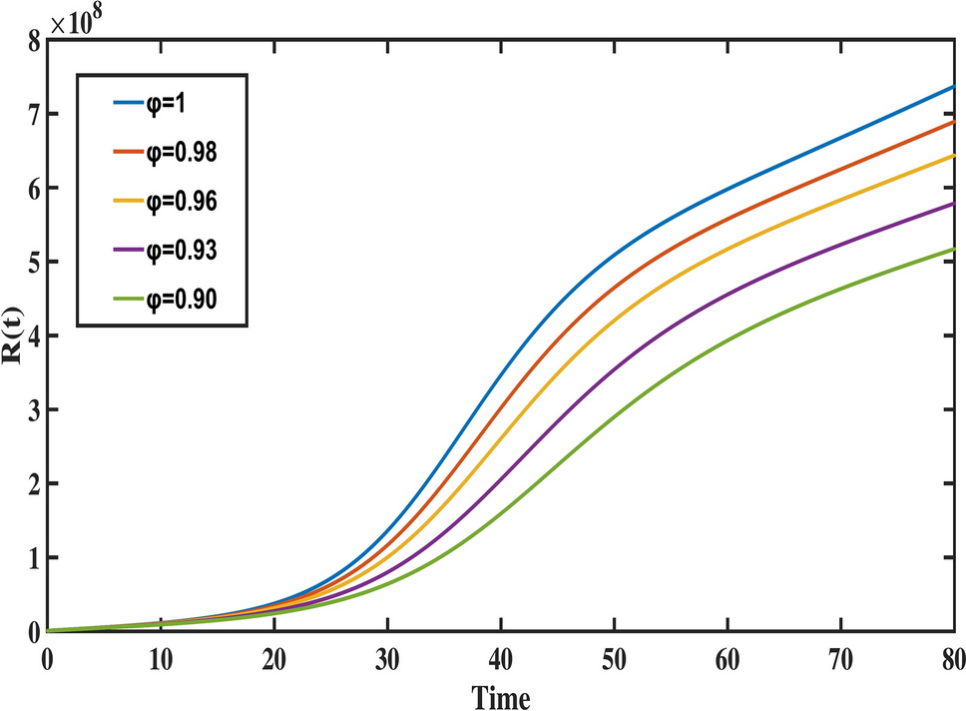
The behavior of recovered population for different values of fractional order ($\varphi$).

To analyze the dynamics of the proposed fractional-order TB model, we graphically represent the behavior of each compartment across various fractional-order values. These visualizations, presented in Figs. 8, 9, 10, 11, 12 and 13, illustrate the impact of the Caputo fractional derivative on the population dynamics under different scenarios: the classical case $\varphi = 1$, fractional cases $\varphi = 0.98$, $\varphi = 0.96$, $\varphi = 0.93$ (fitted to data), and $\varphi = 0.90.$ These graphs allow us to analyze the disease dynamics across these fractional orders and explore the future course.

**Fig. 14:**
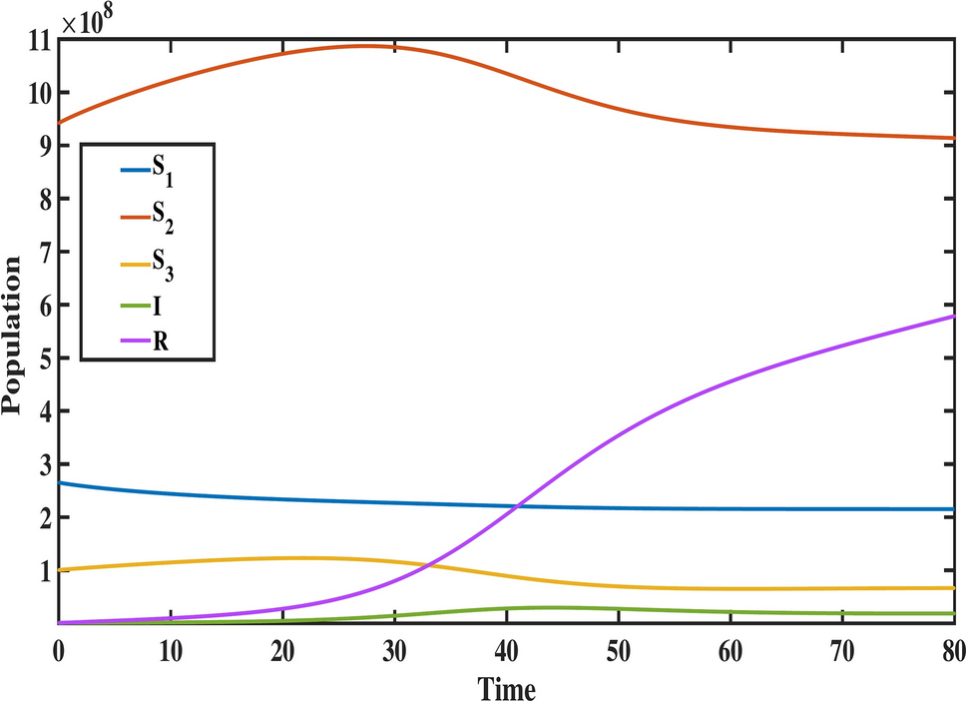
The behavior of all state variables of the model for fractional order ($\varphi =0.93$).

Figure 8 highlights the temporal progression of the younger susceptible population. The graph reveals a sharp initial decline followed by a gradual slowdown, with the curve flattening towards the end. This trend indicates that the model predicts a decrease in the younger population transitioning from susceptibility to exposure over time. Figures 9 and 10 depict the dynamics of the middle-aged and senior susceptible populations, respectively. Both show an initial increase, followed by consistent growth and a gradual decrease after $t=35$. This indicates that the number of middle-aged and senior individuals susceptible to exposure initially increases, followed by a period of stabilization and a gradual decline over time. Notably, the graphs reveal that the Bacillus Calmette-Guerin (BCG) vaccine ($\phi$) is more effective for younger populations and has limited impact on middle-aged and senior individuals, calling for tailored vaccination strategies for these age groups. Figure 12 demonstrates the numerical simulations for the infected compartment. The classical case ($\varphi = 1$) predicts a rapid increase in infections, whereas fractional-order cases show slower infection growth and a more gradual decline post-peak. Similarly, Fig. 11 illustrates the evolution of the exposed population. The trend across different fractional orders aligns with the infected population, where fractional models predict smoother transitions and reduced peak values compared to the integer-order case. The dynamics of the recovered population depicted in Fig. 13, show that fractional-order cases predict an initial slow rise in recoveries, with a substantial increase beginning around $t=20$ and stabilizing after $t=40$. This behavior underscores the model’s ability to represent recovery processes accurately, highlighting the delayed yet significant impact of interventions in reducing the number of infected individuals. Finally, Fig. 14 provides a comprehensive comparison of all compartments for the data-fitted fractional-order value $\varphi = 0.93$. All these results confirm that as $\varphi$ decreases, solutions converge more slowly, and peak values reduce. This behavior indicates the incorporation of memory effects in fractional-order models, making them superior to classical models in capturing the persistence and gradual attenuation of disease dynamics.

## Conclusion

Epidemiological models serve as an essential tool in visualizing the transmission dynamics of diseases, fitting them to real data, and suggesting better control interventions based on thorough analysis. In this study, we formulated a compartmental model to capture the spread dynamics of tuberculosis within a fractional framework to incorporate memory effects. The model employed the Caputo fractional derivative and was validated using real data from China. The key findings and recommendations from the study are outlined below:

- The non-negativity of solutions and the boundedness of the model’s compartments were ensured, highlighting the biological significance of the system. Furthermore, the existence and uniqueness of solutions were rigorously proven, establishing the mathematical robustness of the proposed model.
- The optimal fractional derivative order $(\varphi =0.93)$ obtained through parameter estimation confirms the presence of memory effects in TB transmission, which integer-order models fail to capture. This result supports the use of fractional calculus in epidemiological modeling, as it allows for a more realistic representation of disease dynamics by incorporating long-term dependencies and non-local effects.
- Sensitivity analysis of the basic reproduction number (${\mathscr {R}}_{0}=1.291$) was performed to quantify the impact of model parameters on disease transmission. Results indicated that parameters such as $\gamma _{3}$ (senior infection rate), *r* (recovery rate), and $m_3$ (senior mortality rate) significantly influence ${\mathscr {R}}_{0}$. Additionally, 3D mesh and contour plots were used to visualize the interplay between these parameters and their influence on ${\mathscr {R}}_{0}$, providing actionable insights for intervention strategies.
- The fractional-order model $(\varphi =0.93)$ achieved an RMSE of $5.89e+05$, demonstrating a 28.6% reduction in error compared to the integer-order model (RMSE = $8.24e+055$). This confirms that the fractional-order model provides a significantly better fit to real TB data, reinforcing its superiority in accurately capturing disease dynamics.
- Numerical simulations using the Adams-Bashforth-Moulton method were conducted to analyze the behavior of all compartments under varying fractional orders. These simulations provided valuable insights into disease progression and intervention effectiveness over time.

While Caputo fractional differentiation and integration were used in this study to capture the complexities of tuberculosis dynamics, future research can explore alternative and more advanced fractional operators, such as Caputo-Fabrizio, and Atangana-Baleanu derivatives. Additionally, other datasets can be incorporated to generalize the model’s applicability to different types of biological models. Advanced parameter estimation methods, such as maximum likelihood, can also be considered to improve accuracy further.

## Data Availability

The data utilized in this study were obtained from,^35^ as referenced in the manuscript. Additionally, the data are available upon reasonable request from the second author, Sangeeta Kumawat (00sangeetakumawat@gmail.com).
